# Supplementary material for: Transcriptome Analysis and Identification of Lipid Genes in Physaria lindheimeri, a Genetic Resource for Hydroxy Fatty Acids in Seed Oil
Source: Int J Mol Sci. 2021 Jan 6;22(2):514. doi: 10.3390/ijms22020514 (PMC7825617; doi:10.3390/ijms22020514)
Supplement: Supplementary file 1 [file ijms-22-00514-s001.zip › reiviosin ijms-1021173 Sup files_KHU and Chen/Sup file 1, Figure S1, Figure S2.pptx]

## Slide 1
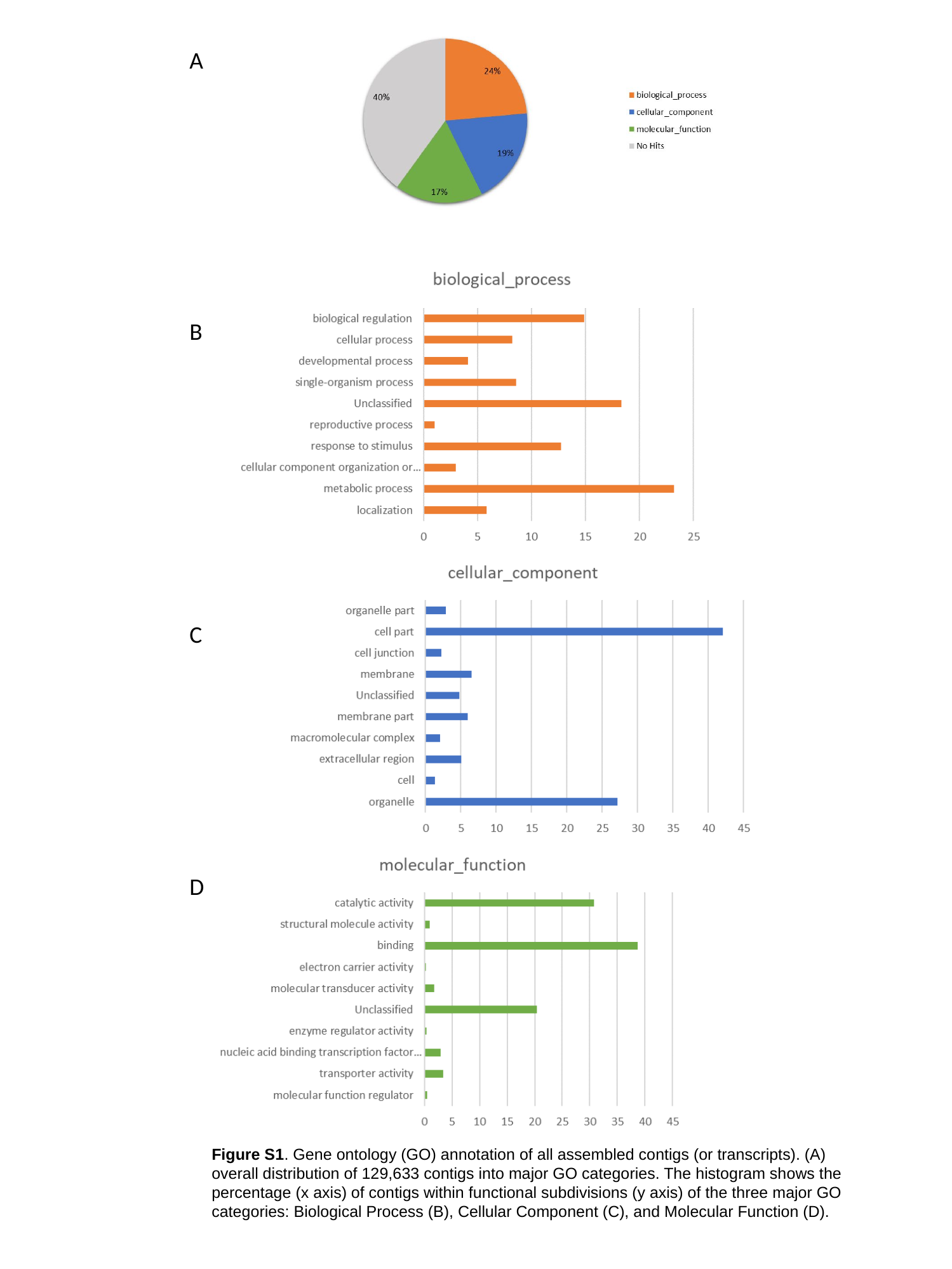

A
B
C
D
Figure S1. Gene ontology (GO) annotation of all assembled contigs (or transcripts). (A) overall distribution of 129,633 contigs into major GO categories. The histogram shows the percentage (x axis) of contigs within functional subdivisions (y axis) of the three major GO categories: Biological Process (B), Cellular Component (C), and Molecular Function (D).

## Slide 2
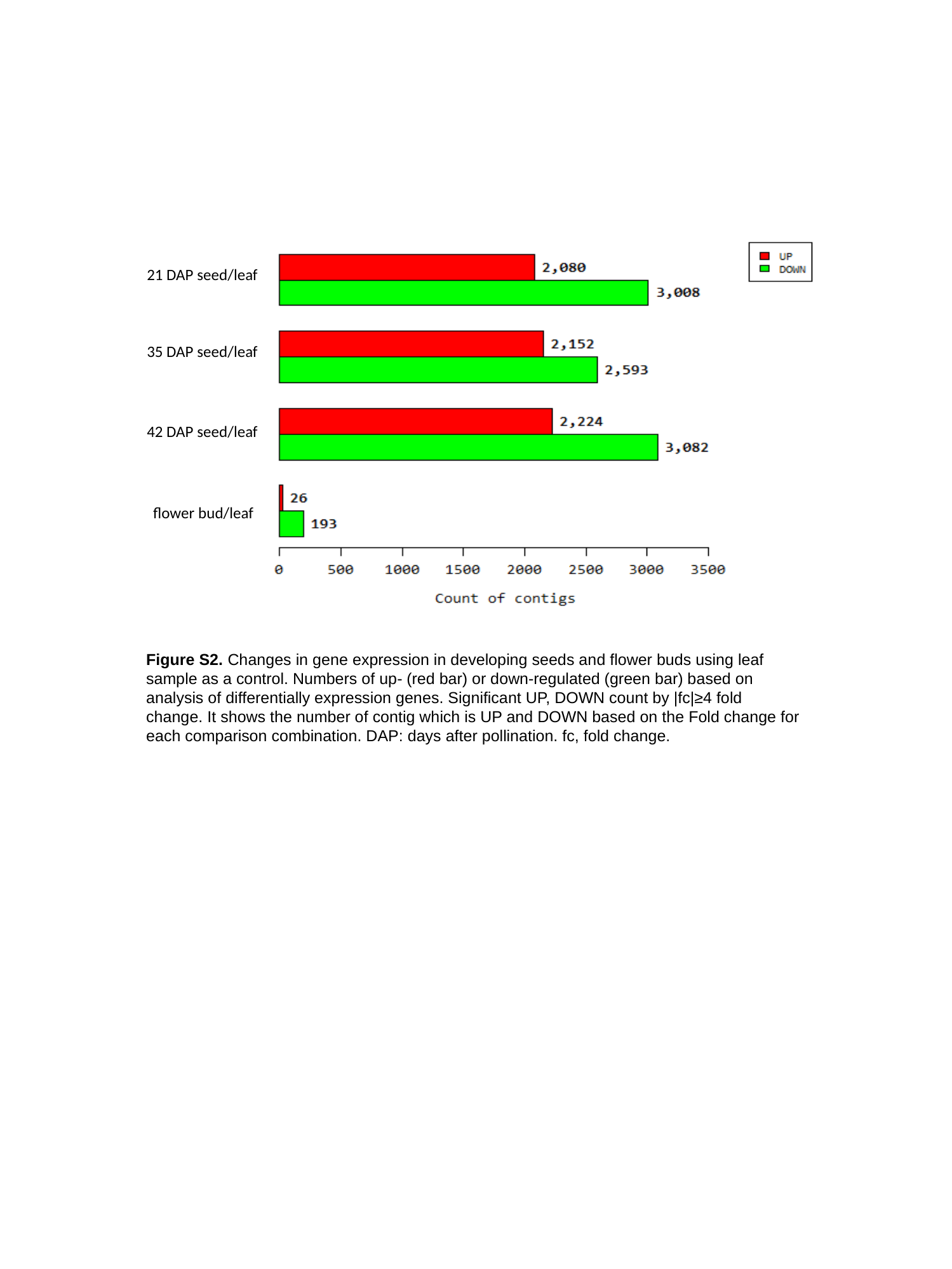

21 DAP seed/leaf
35 DAP seed/leaf
42 DAP seed/leaf
flower bud/leaf
Figure S2. Changes in gene expression in developing seeds and flower buds using leaf sample as a control. Numbers of up- (red bar) or down-regulated (green bar) based on analysis of differentially expression genes. Significant UP, DOWN count by |fc|≥4 fold change. It shows the number of contig which is UP and DOWN based on the Fold change for each comparison combination. DAP: days after pollination. fc, fold change.
