# Supplementary material for: Transcriptome Analysis and Identification of Lipid Genes in Physaria lindheimeri, a Genetic Resource for Hydroxy Fatty Acids in Seed Oil
Source: Int J Mol Sci. 2021 Jan 6;22(2):514. doi: 10.3390/ijms22020514 (PMC7825617; doi:10.3390/ijms22020514)
Supplement: Supplementary file 1 [file ijms-22-00514-s001.zip › reiviosin ijms-1021173 Sup files_KHU and Chen/Sup file 10, Figure S10.pptx]

## Slide 1
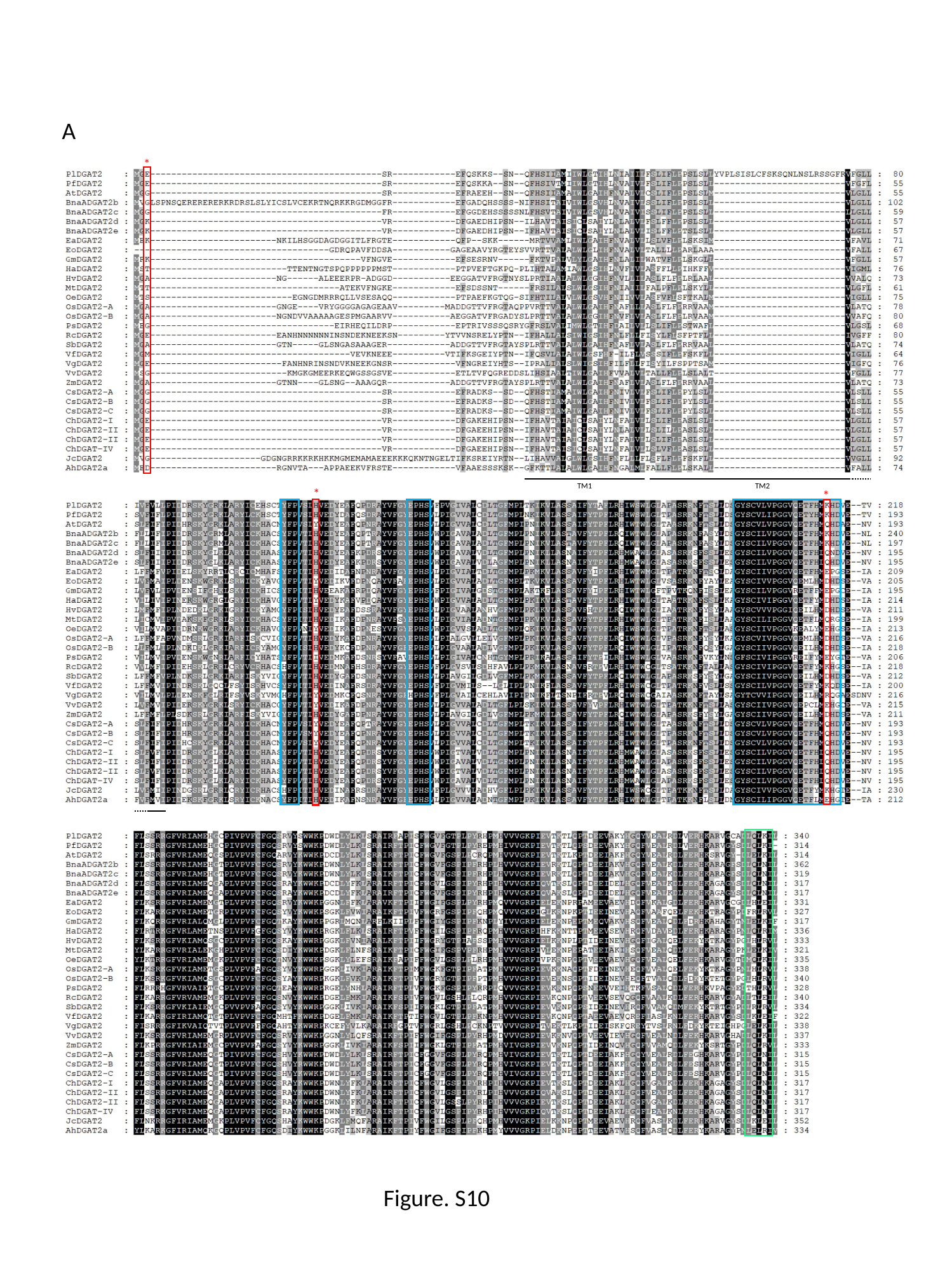

A
*
TM1
TM2
*
*
Figure. S10

## Slide 2
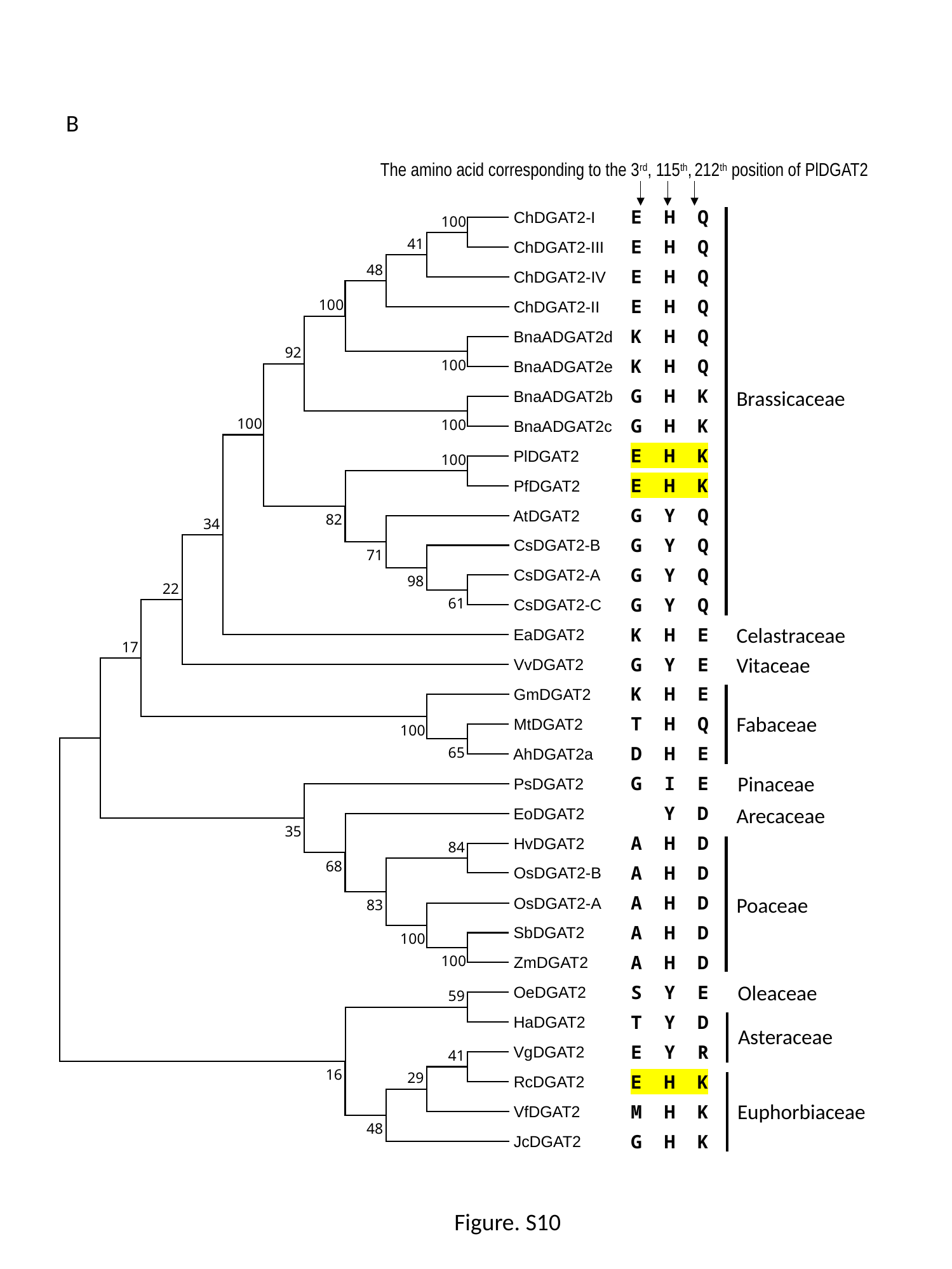

B
The amino acid corresponding to the 3rd, 115th, 212th position of PlDGAT2
 ChDGAT2-I
100
41
 ChDGAT2-III
48
 ChDGAT2-IV
100
 ChDGAT2-II
 BnaADGAT2d
92
100
 BnaADGAT2e
 BnaADGAT2b
100
100
 BnaADGAT2c
 PlDGAT2
100
 PfDGAT2
 AtDGAT2
82
34
 CsDGAT2-B
71
 CsDGAT2-A
98
22
61
 CsDGAT2-C
 EaDGAT2
17
 VvDGAT2
 GmDGAT2
 MtDGAT2
100
65
 AhDGAT2a
 PsDGAT2
 EoDGAT2
35
 HvDGAT2
84
68
 OsDGAT2-B
 OsDGAT2-A
83
 SbDGAT2
100
100
 ZmDGAT2
 OeDGAT2
59
 HaDGAT2
 VgDGAT2
41
16
29
 RcDGAT2
 VfDGAT2
48
 JcDGAT2
 E H Q
 E H Q
 E H Q
 E H Q
 K H Q
 K H Q
 G H K
 G H K
 E H K
 E H K
 G Y Q
 G Y Q
 G Y Q
 G Y Q
 K H E
 G Y E
 K H E
 T H Q
 D H E
 G I E
 A Y D
 A H D
 A H D
 A H D
 A H D
 A H D
 S Y E
 T Y D
 E Y R
 E H K
 M H K
 G H K
Brassicaceae
Celastraceae
Vitaceae
Fabaceae
Pinaceae
Arecaceae
Poaceae
Oleaceae
Asteraceae
Euphorbiaceae
Figure. S10

## Slide 3
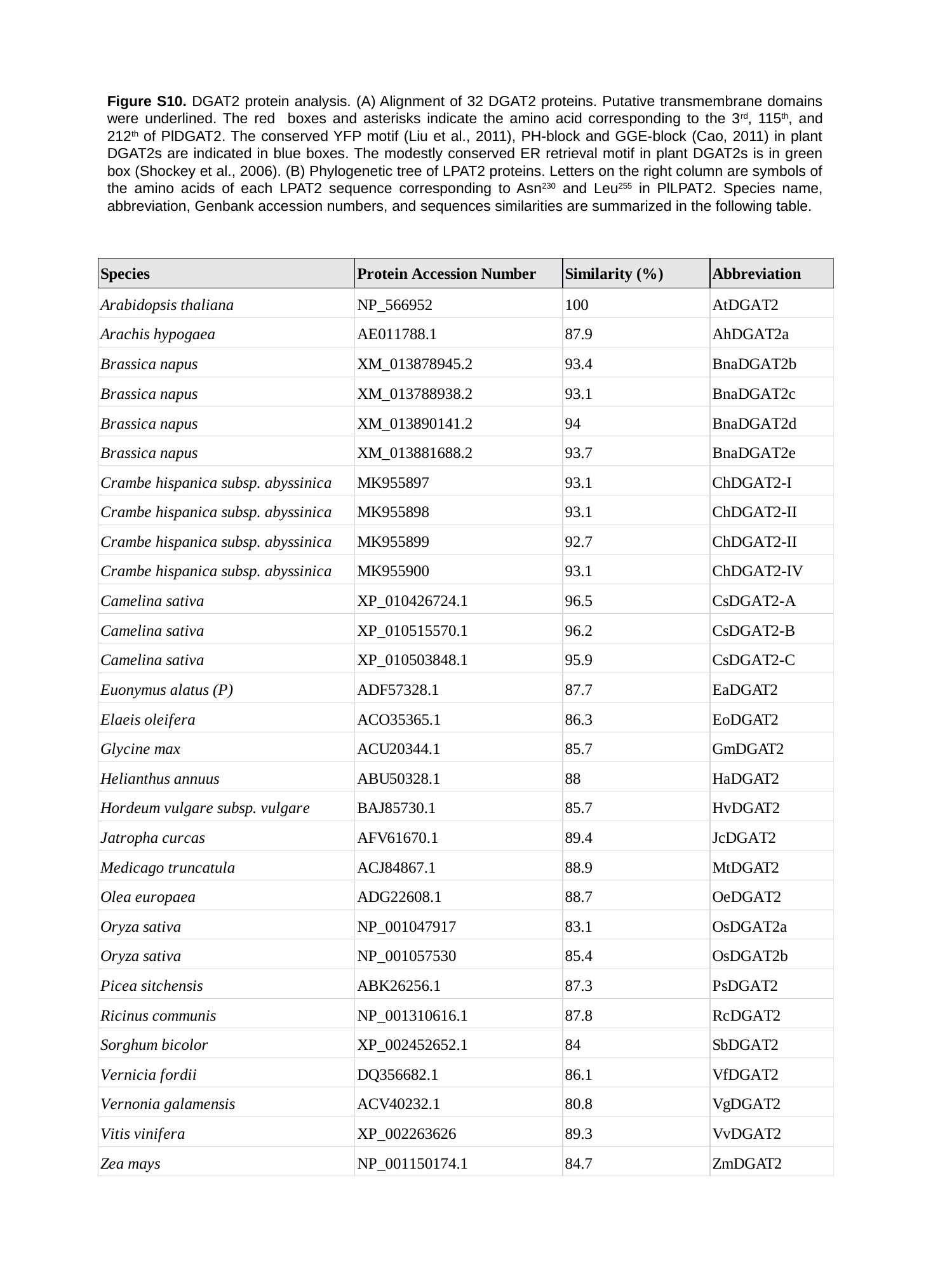

Figure S10. DGAT2 protein analysis. (A) Alignment of 32 DGAT2 proteins. Putative transmembrane domains were underlined. The red boxes and asterisks indicate the amino acid corresponding to the 3rd, 115th, and 212th of PlDGAT2. The conserved YFP motif (Liu et al., 2011), PH-block and GGE-block (Cao, 2011) in plant DGAT2s are indicated in blue boxes. The modestly conserved ER retrieval motif in plant DGAT2s is in green box (Shockey et al., 2006). (B) Phylogenetic tree of LPAT2 proteins. Letters on the right column are symbols of the amino acids of each LPAT2 sequence corresponding to Asn230 and Leu255 in PlLPAT2. Species name, abbreviation, Genbank accession numbers, and sequences similarities are summarized in the following table.
