# Supplementary material for: Transcriptome Analysis and Identification of Lipid Genes in Physaria lindheimeri, a Genetic Resource for Hydroxy Fatty Acids in Seed Oil
Source: Int J Mol Sci. 2021 Jan 6;22(2):514. doi: 10.3390/ijms22020514 (PMC7825617; doi:10.3390/ijms22020514)
Supplement: Supplementary file 1 [file ijms-22-00514-s001.zip › reiviosin ijms-1021173 Sup files_KHU and Chen/Sup file 11, Figure S11.pptx]

## Slide 1
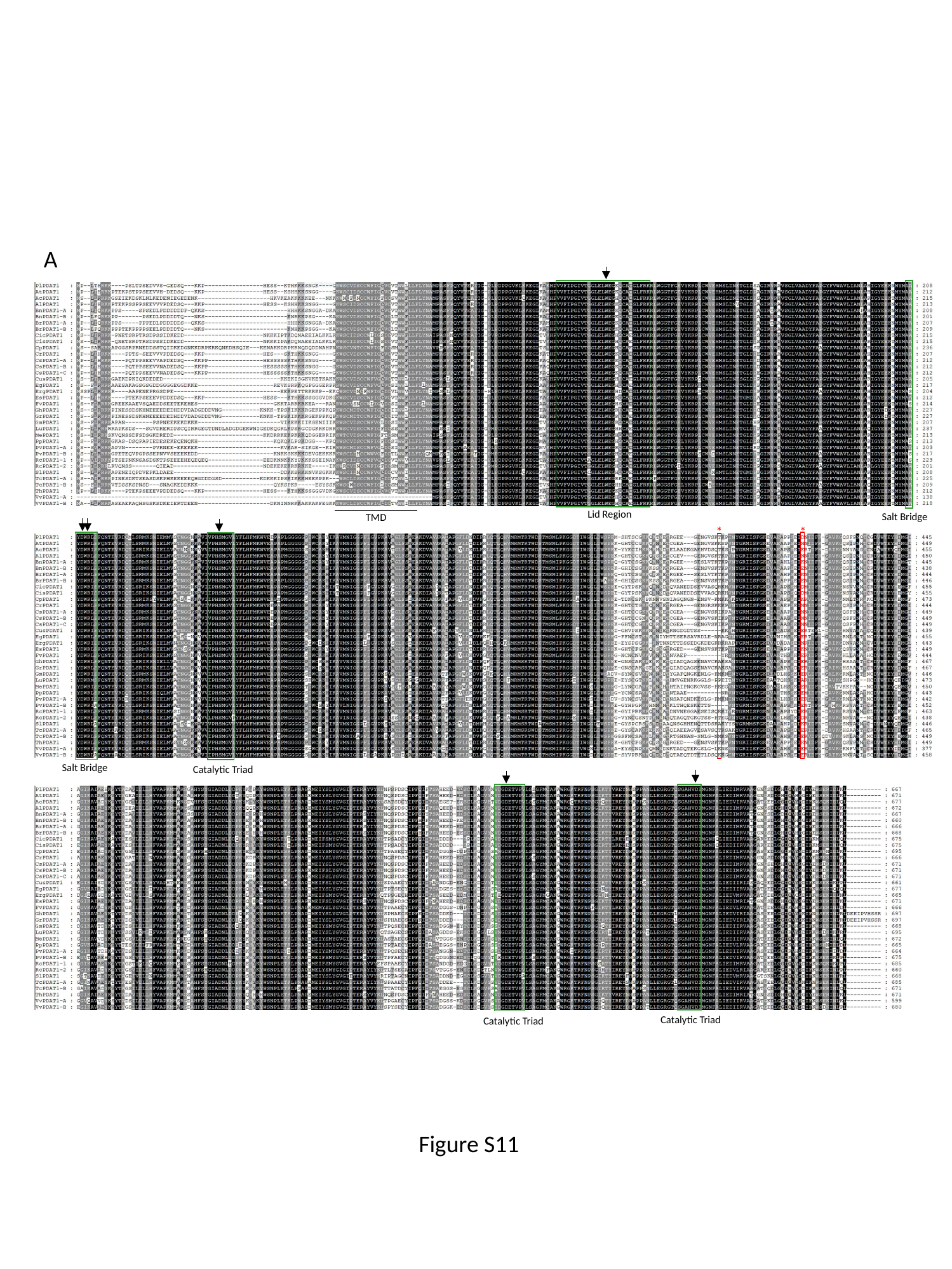

A
Lid Region
Salt Bridge
TMD
*
*
Salt Bridge
Catalytic Triad
Catalytic Triad
Catalytic Triad
Figure S11

## Slide 2
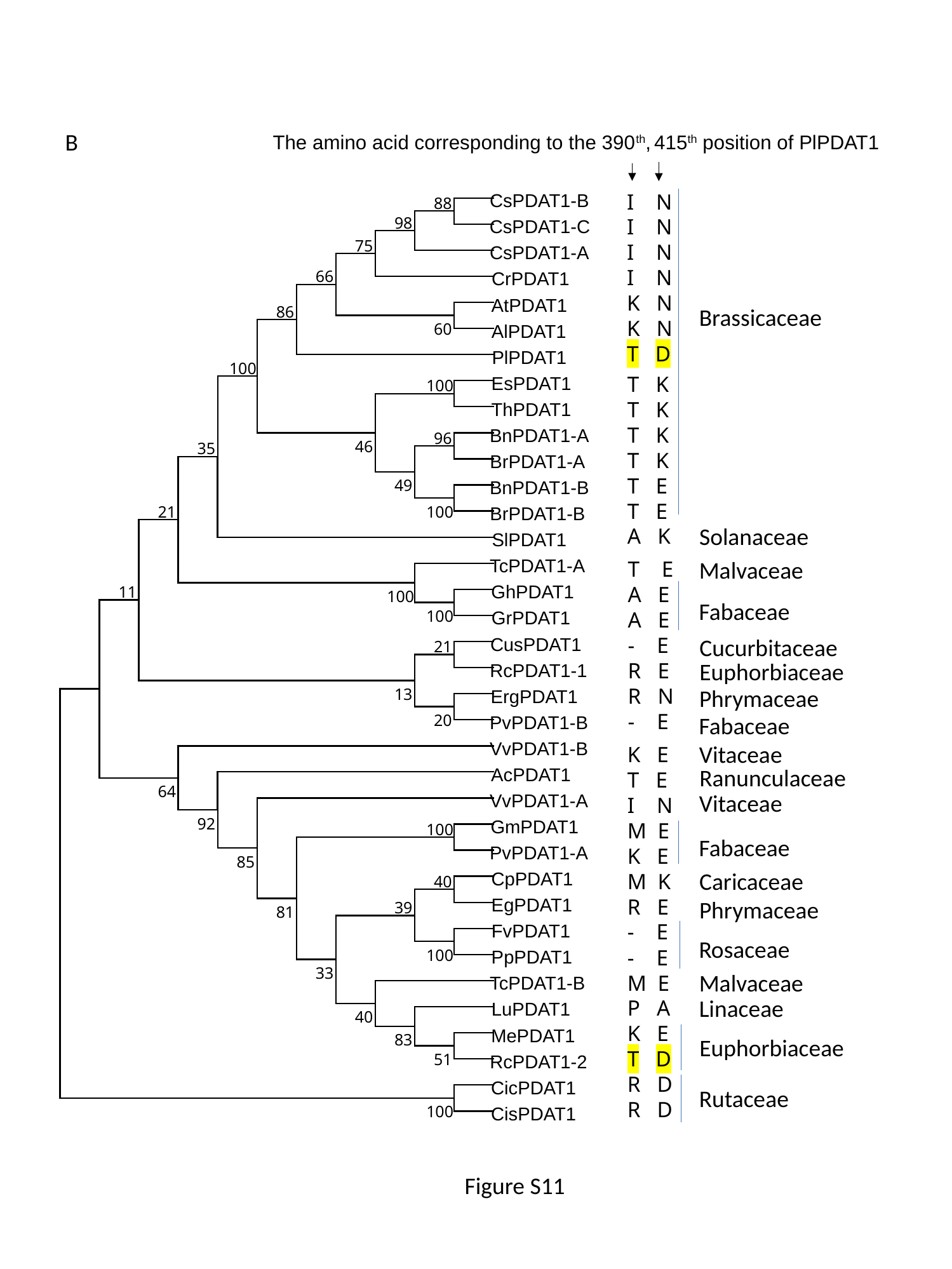

B
The amino acid corresponding to the 390th, 415th position of PlPDAT1
 CsPDAT1-B
88
98
 CsPDAT1-C
75
 CsPDAT1-A
66
 CrPDAT1
 AtPDAT1
86
60
 AlPDAT1
 PlPDAT1
100
 EsPDAT1
100
 ThPDAT1
 BnPDAT1-A
96
46
35
 BrPDAT1-A
49
 BnPDAT1-B
21
100
 BrPDAT1-B
 SlPDAT1
 TcPDAT1-A
 GhPDAT1
11
100
100
 GrPDAT1
 CusPDAT1
21
 RcPDAT1-1
13
 ErgPDAT1
20
 PvPDAT1-B
 VvPDAT1-B
 AcPDAT1
64
 VvPDAT1-A
92
 GmPDAT1
100
 PvPDAT1-A
85
 CpPDAT1
40
 EgPDAT1
39
81
 FvPDAT1
100
 PpPDAT1
33
 TcPDAT1-B
 LuPDAT1
40
 MePDAT1
83
51
 RcPDAT1-2
 CicPDAT1
100
 CisPDAT1
I N
I N
I N
I N
K N
K N
T D
Brassicaceae
T K
T K
T K
T K
T E
T E
A K
Solanaceae
Malvaceae
T E
A E
A E
- E
R E
R N
- E
Fabaceae
Cucurbitaceae
Euphorbiaceae
Phrymaceae
Fabaceae
Vitaceae
K E
T E
I N
M E
K E
M K
R E
- E
- E
M E
P A
K E
T D
R D
R D
Ranunculaceae
Vitaceae
Fabaceae
Caricaceae
Phrymaceae
Rosaceae
Malvaceae
Linaceae
Euphorbiaceae
Rutaceae
Figure S11

## Slide 3
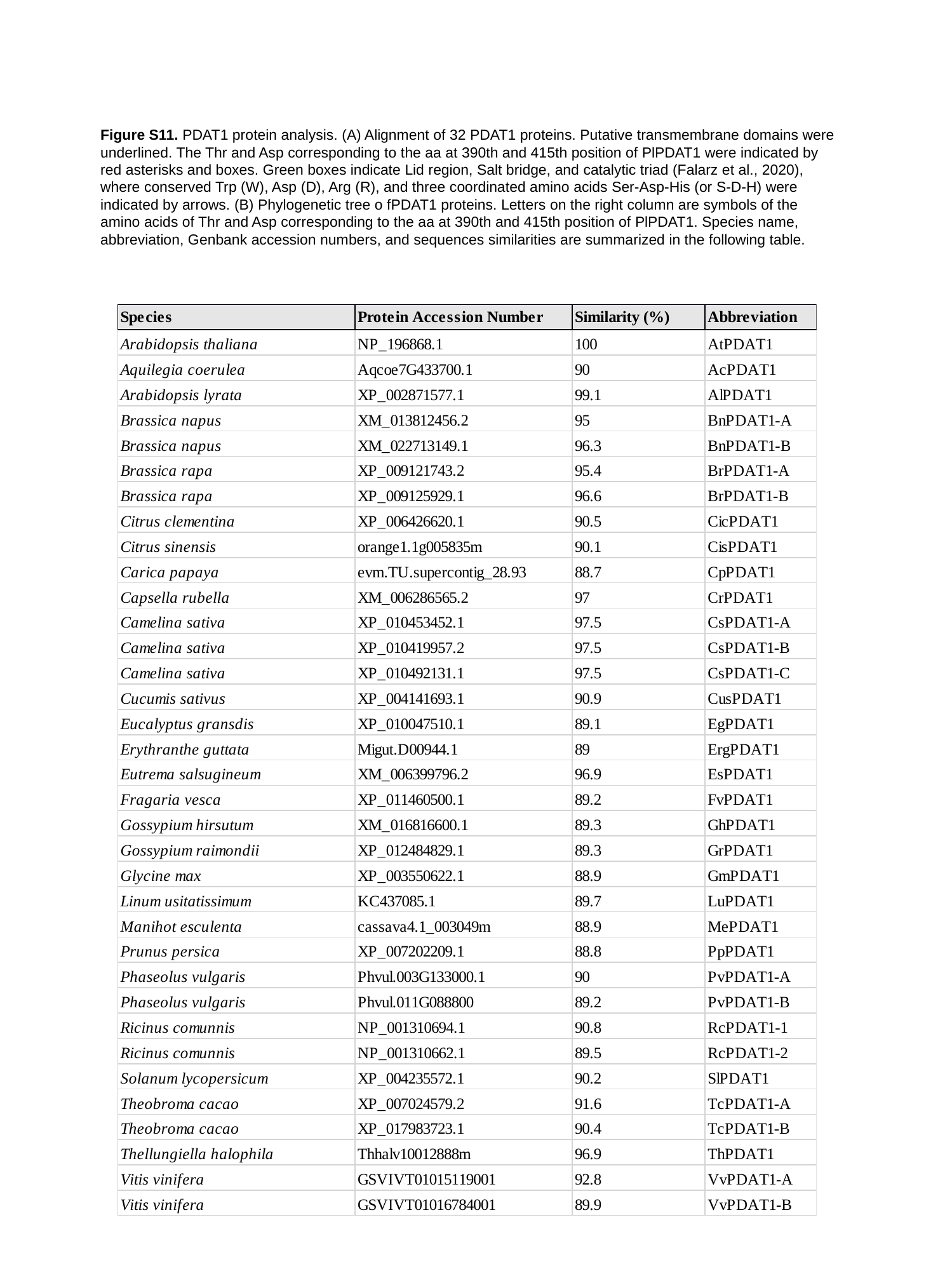

Figure S11. PDAT1 protein analysis. (A) Alignment of 32 PDAT1 proteins. Putative transmembrane domains were underlined. The Thr and Asp corresponding to the aa at 390th and 415th position of PlPDAT1 were indicated by red asterisks and boxes. Green boxes indicate Lid region, Salt bridge, and catalytic triad (Falarz et al., 2020), where conserved Trp (W), Asp (D), Arg (R), and three coordinated amino acids Ser-Asp-His (or S-D-H) were indicated by arrows. (B) Phylogenetic tree o fPDAT1 proteins. Letters on the right column are symbols of the amino acids of Thr and Asp corresponding to the aa at 390th and 415th position of PlPDAT1. Species name, abbreviation, Genbank accession numbers, and sequences similarities are summarized in the following table.
