# Supplementary material for: Transcriptome Analysis and Identification of Lipid Genes in Physaria lindheimeri, a Genetic Resource for Hydroxy Fatty Acids in Seed Oil
Source: Int J Mol Sci. 2021 Jan 6;22(2):514. doi: 10.3390/ijms22020514 (PMC7825617; doi:10.3390/ijms22020514)
Supplement: Supplementary file 1 [file ijms-22-00514-s001.zip › reiviosin ijms-1021173 Sup files_KHU and Chen/Sup file 12, Figure S12.pptx]

## Slide 1
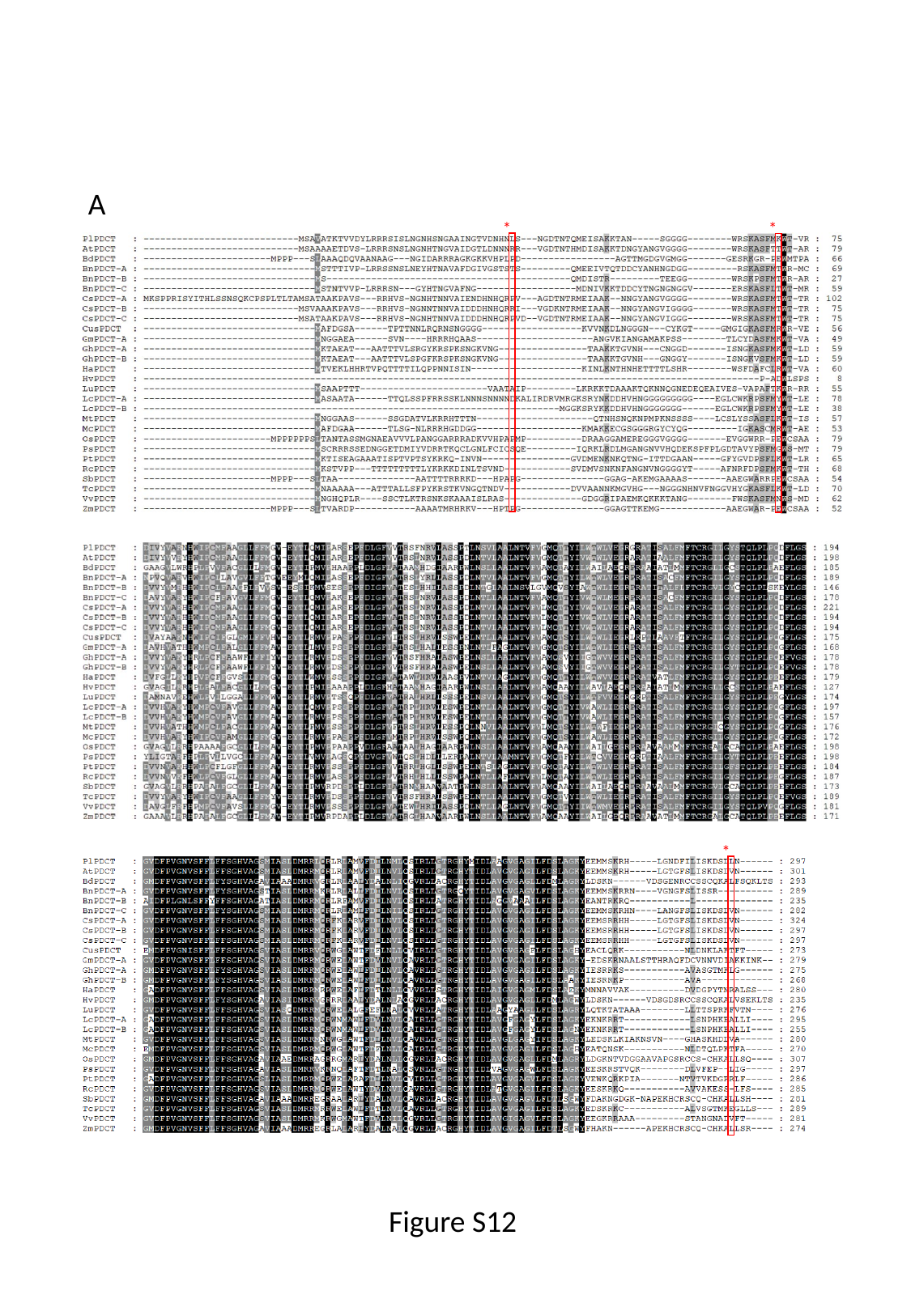

A
*
*
*
Figure S12

## Slide 2
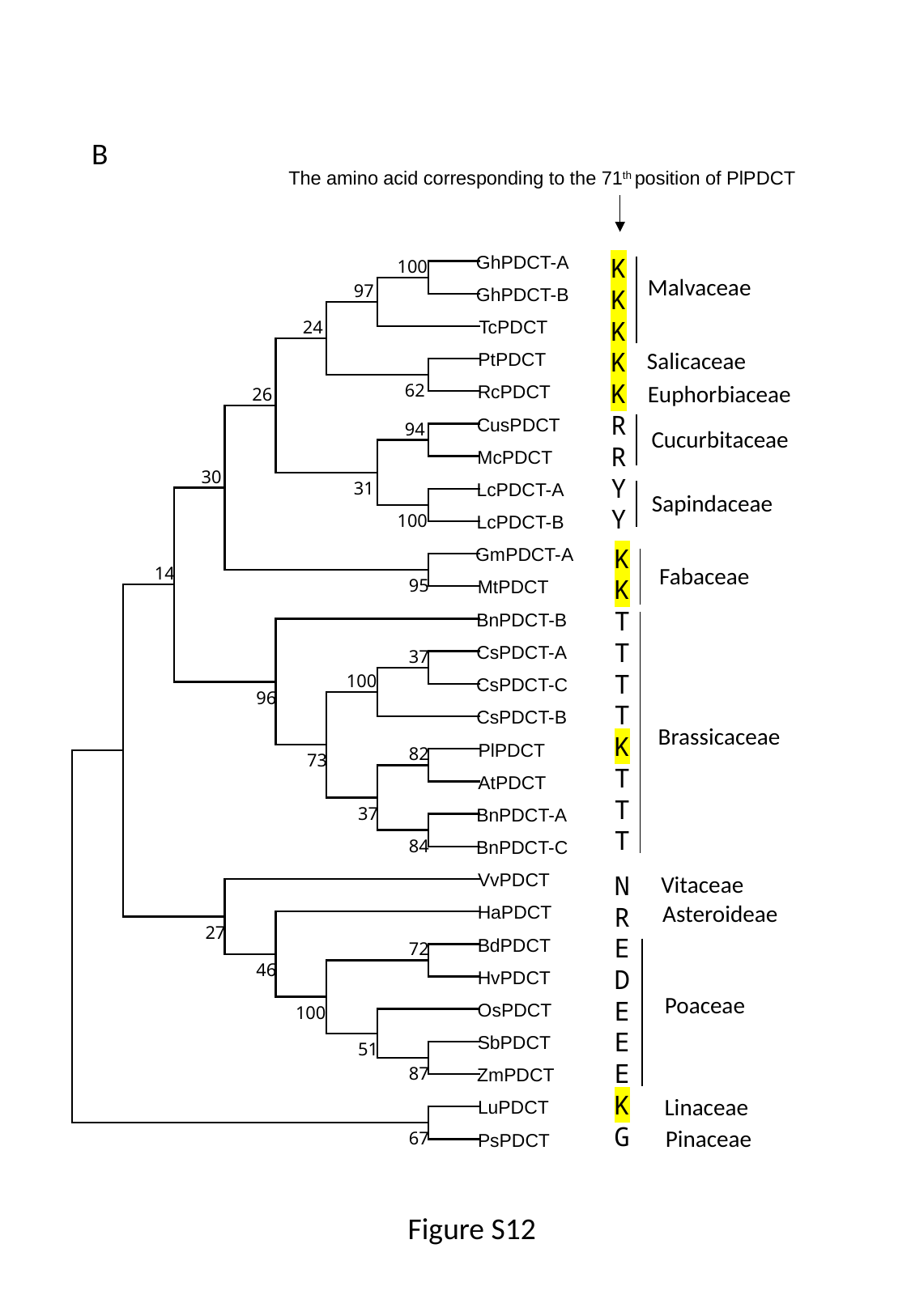

B
The amino acid corresponding to the 71th position of PlPDCT
 GhPDCT-A
100
97
 GhPDCT-B
 TcPDCT
24
 PtPDCT
62
 RcPDCT
26
 CusPDCT
94
 McPDCT
30
31
 LcPDCT-A
100
 LcPDCT-B
 GmPDCT-A
14
95
 MtPDCT
 BnPDCT-B
 CsPDCT-A
37
100
 CsPDCT-C
96
 CsPDCT-B
 PlPDCT
82
73
 AtPDCT
37
 BnPDCT-A
84
 BnPDCT-C
 VvPDCT
 HaPDCT
27
 BdPDCT
72
46
 HvPDCT
 OsPDCT
100
 SbPDCT
51
87
 ZmPDCT
 LuPDCT
67
 PsPDCT
K
K
K
K
K
R
R
Y
Y
Malvaceae
Salicaceae
Euphorbiaceae
Cucurbitaceae
Sapindaceae
K
K
T
T
T
T
K
T
T
T
Fabaceae
Brassicaceae
N
R
E
D
E
E
E
K
G
Vitaceae
Asteroideae
Poaceae
Linaceae
Pinaceae
Figure S12

## Slide 3
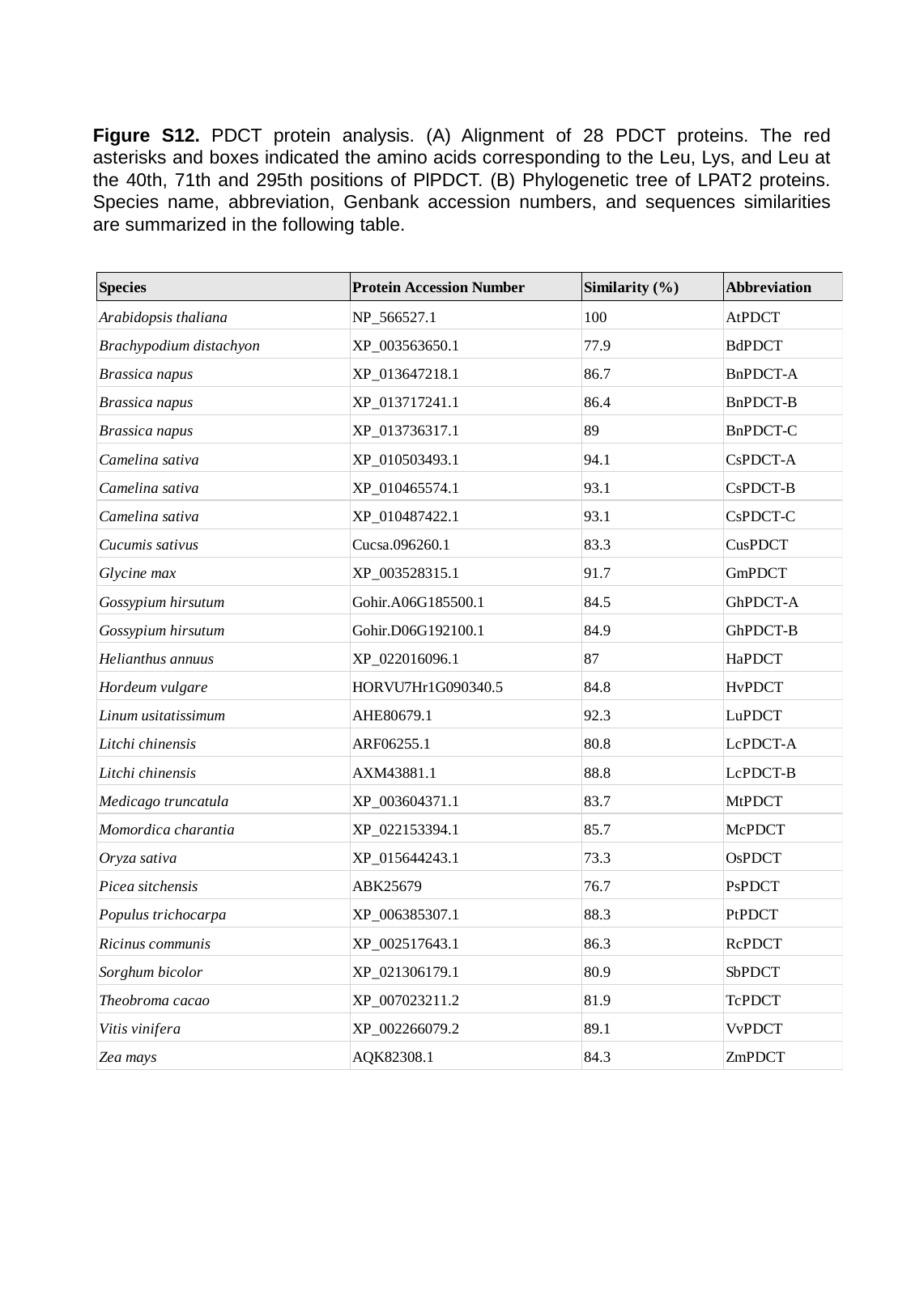

Figure S12. PDCT protein analysis. (A) Alignment of 28 PDCT proteins. The red asterisks and boxes indicated the amino acids corresponding to the Leu, Lys, and Leu at the 40th, 71th and 295th positions of PlPDCT. (B) Phylogenetic tree of LPAT2 proteins. Species name, abbreviation, Genbank accession numbers, and sequences similarities are summarized in the following table.
