# Supplementary material for: Transcriptome Analysis and Identification of Lipid Genes in Physaria lindheimeri, a Genetic Resource for Hydroxy Fatty Acids in Seed Oil
Source: Int J Mol Sci. 2021 Jan 6;22(2):514. doi: 10.3390/ijms22020514 (PMC7825617; doi:10.3390/ijms22020514)
Supplement: Supplementary file 1 [file ijms-22-00514-s001.zip › reiviosin ijms-1021173 Sup files_KHU and Chen/Sup file 13, Figure S13.pptx]

## Slide 1
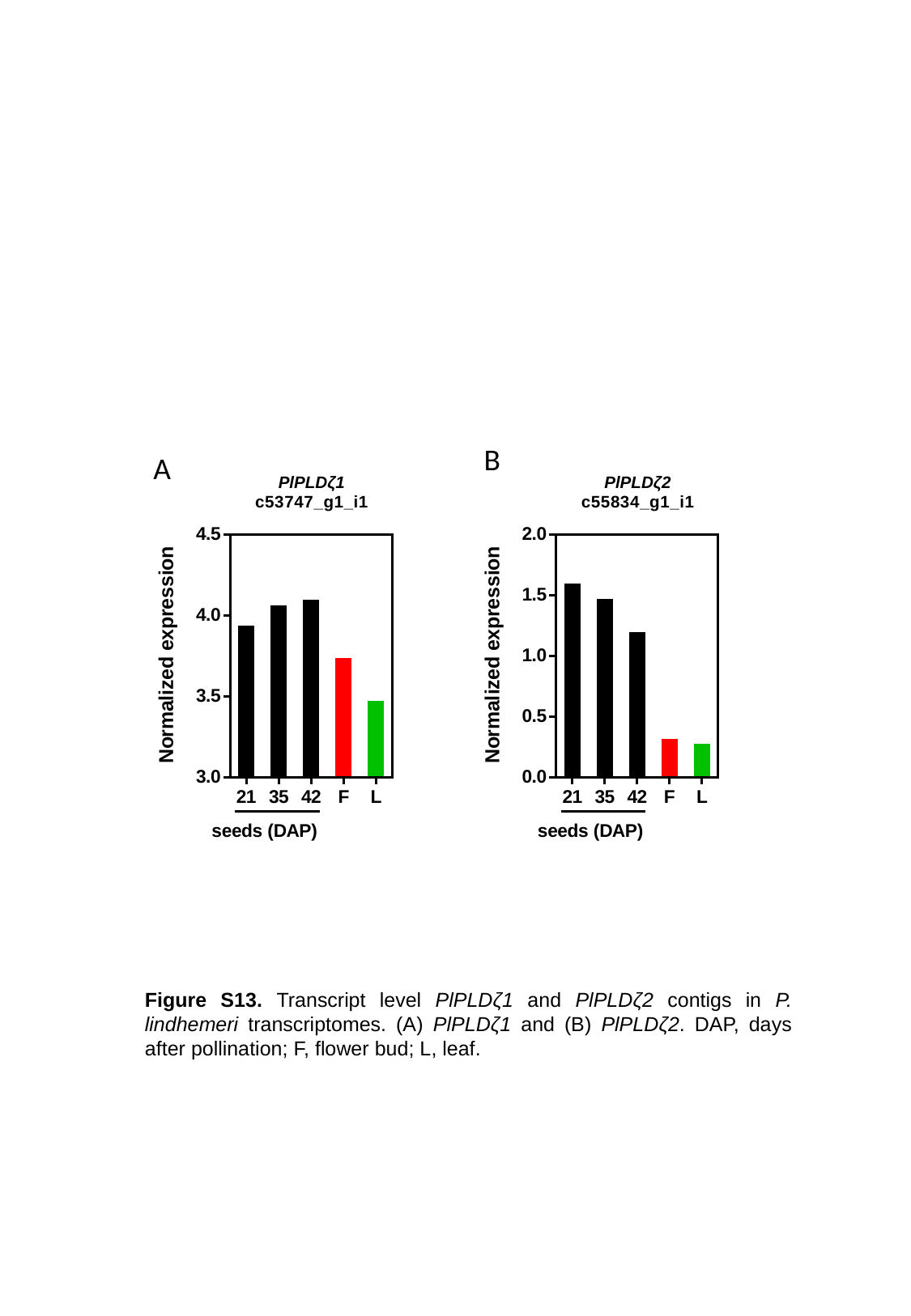

B
A
Figure S13. Transcript level PlPLDζ1 and PlPLDζ2 contigs in P. lindhemeri transcriptomes. (A) PlPLDζ1 and (B) PlPLDζ2. DAP, days after pollination; F, flower bud; L, leaf.
