# Supplementary material for: Transcriptome Analysis and Identification of Lipid Genes in Physaria lindheimeri, a Genetic Resource for Hydroxy Fatty Acids in Seed Oil
Source: Int J Mol Sci. 2021 Jan 6;22(2):514. doi: 10.3390/ijms22020514 (PMC7825617; doi:10.3390/ijms22020514)
Supplement: Supplementary file 1 [file ijms-22-00514-s001.zip › reiviosin ijms-1021173 Sup files_KHU and Chen/Sup file 4, Figure S3 to Figure S6.pptx]

## Slide 1
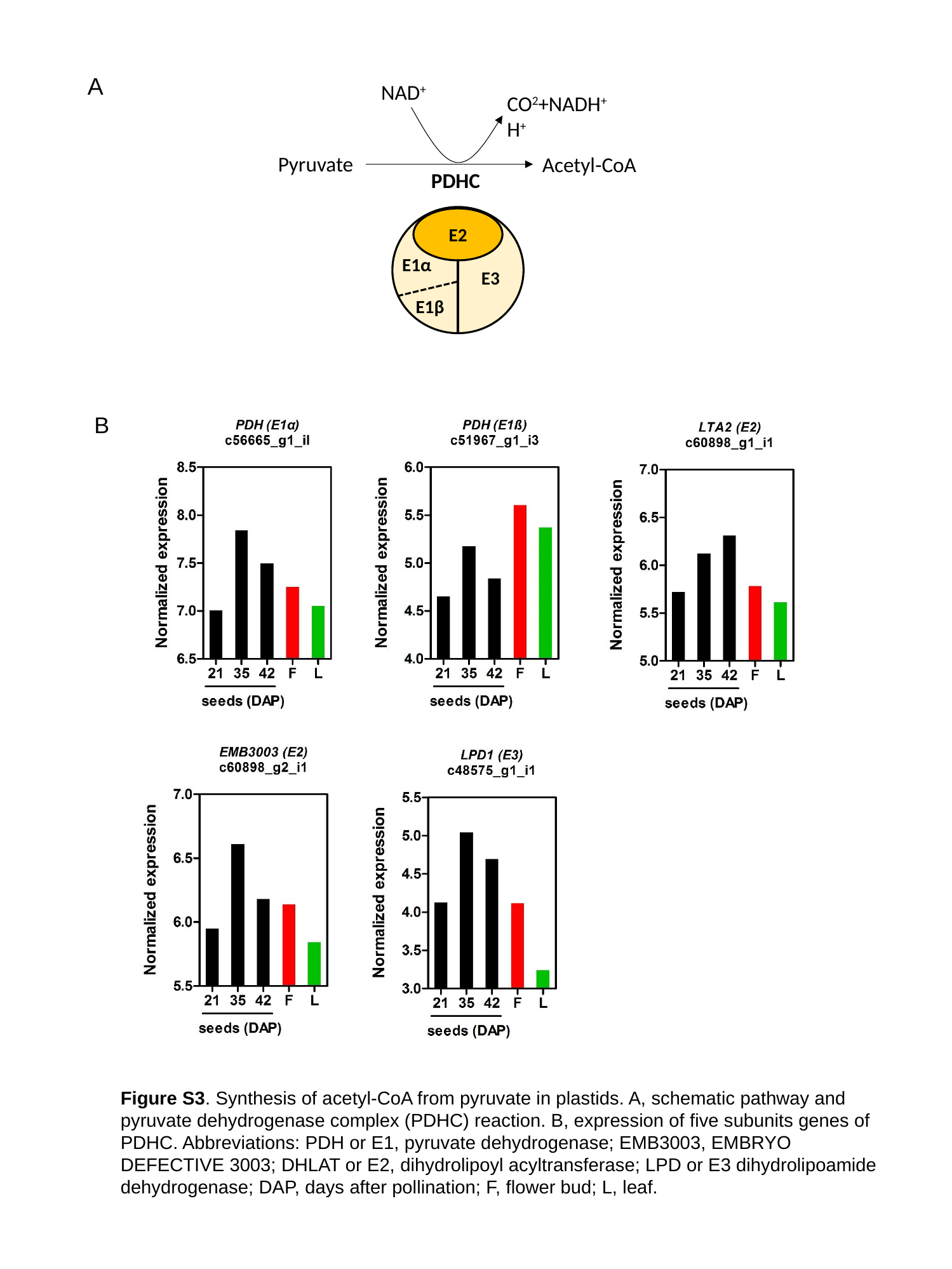

A
NAD+
CO2+NADH+ H+
Pyruvate
Acetyl-CoA
PDHC
E2
E1α
E3
E1β
B
Figure S3. Synthesis of acetyl-CoA from pyruvate in plastids. A, schematic pathway and pyruvate dehydrogenase complex (PDHC) reaction. B, expression of five subunits genes of PDHC. Abbreviations: PDH or E1, pyruvate dehydrogenase; EMB3003, EMBRYO DEFECTIVE 3003; DHLAT or E2, dihydrolipoyl acyltransferase; LPD or E3 dihydrolipoamide dehydrogenase; DAP, days after pollination; F, flower bud; L, leaf.

## Slide 2
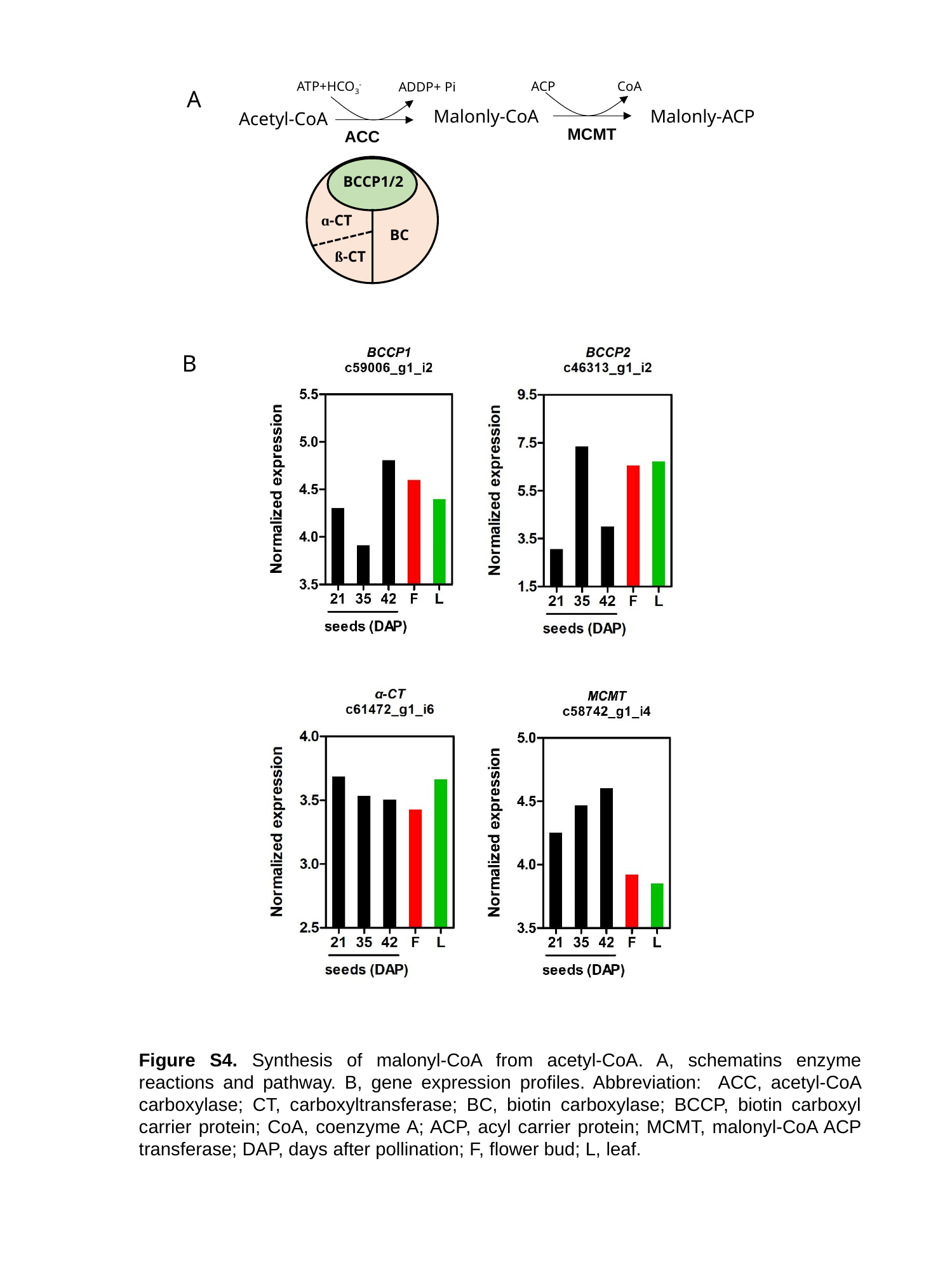

ACP
CoA
ATP+HCO3-
ADDP+ Pi
A
Malonly-CoA
Malonly-ACP
Acetyl-CoA
MCMT
ACC
BCCP1/2
ɑ-CT
BC
ß-CT
B
Figure S4. Synthesis of malonyl-CoA from acetyl-CoA. A, schematins enzyme reactions and pathway. B, gene expression profiles. Abbreviation: ACC, acetyl-CoA carboxylase; CT, carboxyltransferase; BC, biotin carboxylase; BCCP, biotin carboxyl carrier protein; CoA, coenzyme A; ACP, acyl carrier protein; MCMT, malonyl-CoA ACP transferase; DAP, days after pollination; F, flower bud; L, leaf.

## Slide 3
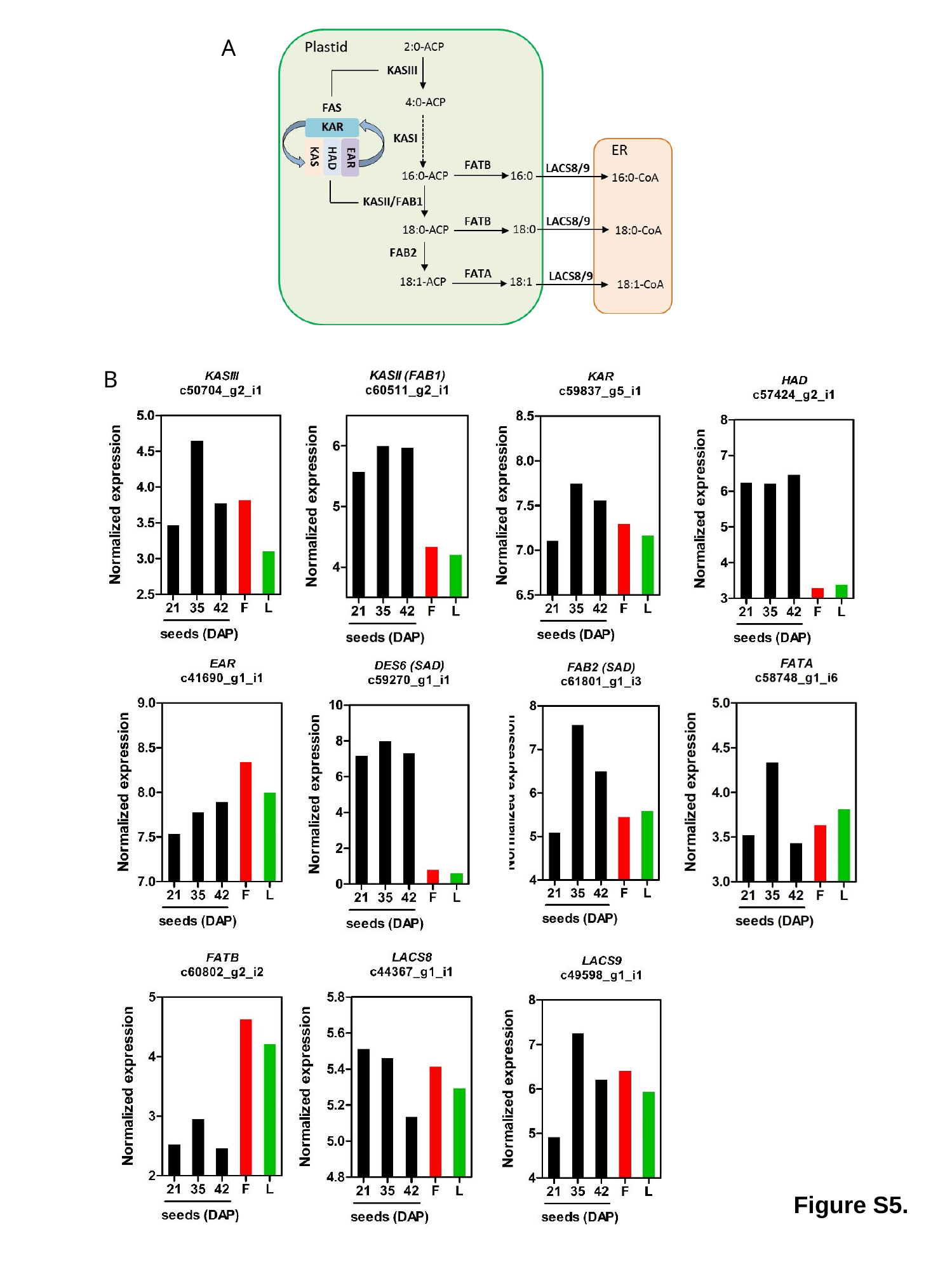

A
B
Figure S5.

## Slide 4
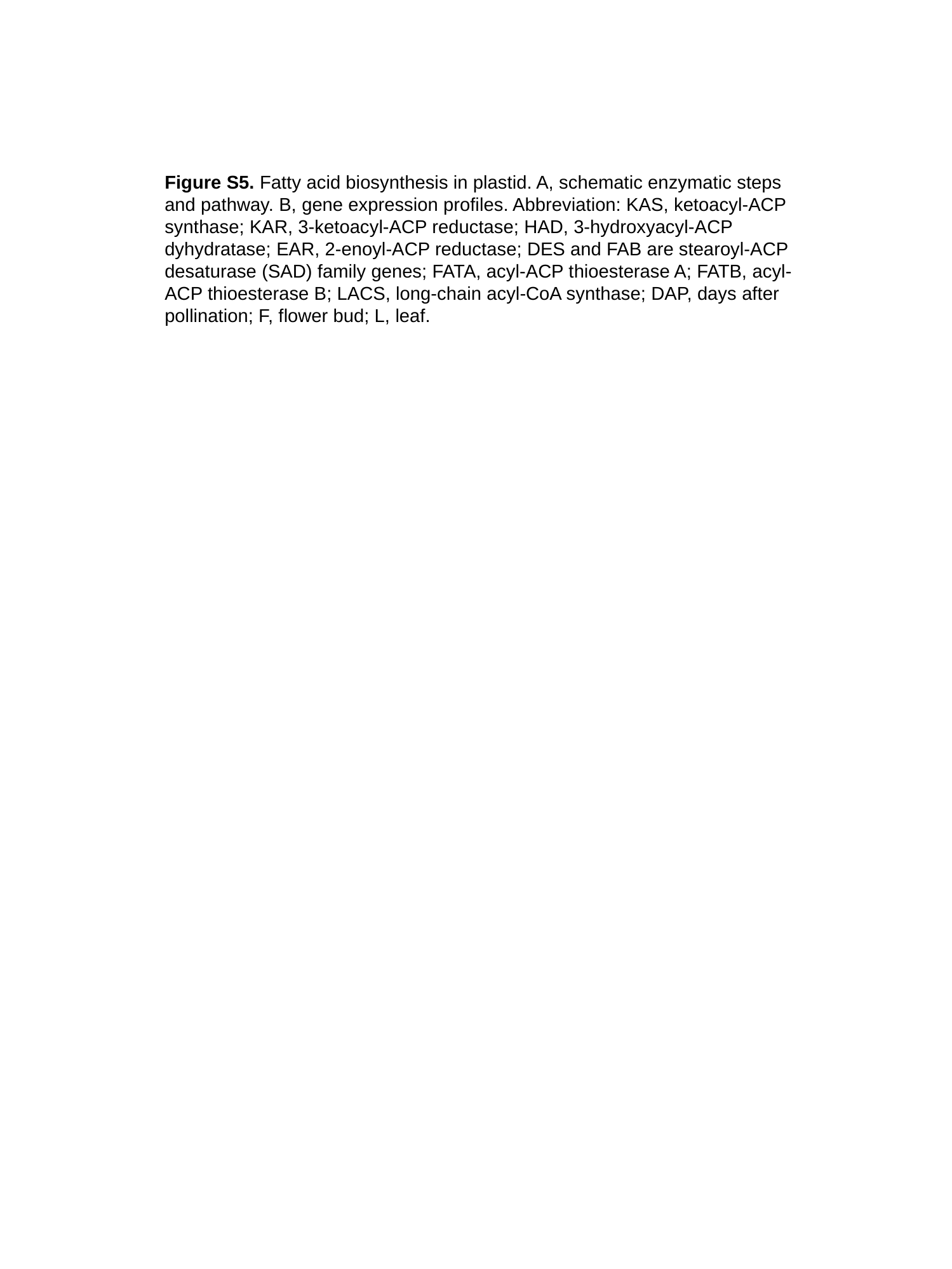

Figure S5. Fatty acid biosynthesis in plastid. A, schematic enzymatic steps and pathway. B, gene expression profiles. Abbreviation: KAS, ketoacyl-ACP synthase; KAR, 3-ketoacyl-ACP reductase; HAD, 3-hydroxyacyl-ACP dyhydratase; EAR, 2-enoyl-ACP reductase; DES and FAB are stearoyl-ACP desaturase (SAD) family genes; FATA, acyl-ACP thioesterase A; FATB, acyl-ACP thioesterase B; LACS, long-chain acyl-CoA synthase; DAP, days after pollination; F, flower bud; L, leaf.

## Slide 5
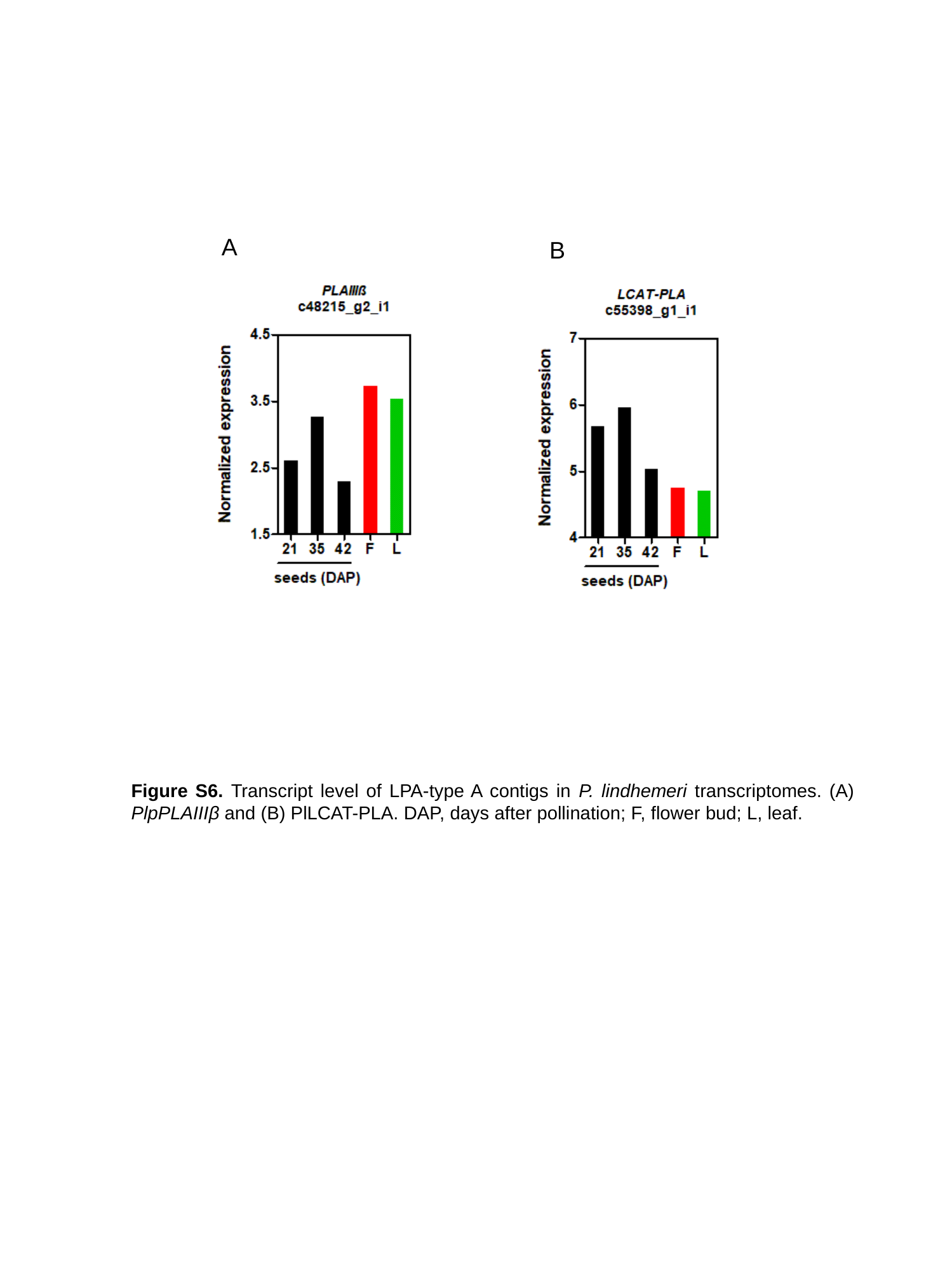

A
B
Figure S6. Transcript level of LPA-type A contigs in P. lindhemeri transcriptomes. (A) PlpPLAIIIβ and (B) PlLCAT-PLA. DAP, days after pollination; F, flower bud; L, leaf.
