# Supplementary material for: Transcriptome Analysis and Identification of Lipid Genes in Physaria lindheimeri, a Genetic Resource for Hydroxy Fatty Acids in Seed Oil
Source: Int J Mol Sci. 2021 Jan 6;22(2):514. doi: 10.3390/ijms22020514 (PMC7825617; doi:10.3390/ijms22020514)
Supplement: Supplementary file 1 [file ijms-22-00514-s001.zip › reiviosin ijms-1021173 Sup files_KHU and Chen/Sup file 5, Method of cDNA cloning.docx]

**Supplementary File 5.** Method of coning full length cDNAs of *PlFAD2* and *PlFAD3-1*

Total RNA was extracted from the developing seed and leaf as described in Materials and Methods. The cDNA was synthesized from mRNA of *Physaria lindheimeri* using the 1st cDNA synthesis kit (Takara, https://www.takarabio.com). To amplify the CDS region of *PlFAD2*, the forward primer was designed as ATGGGTGCAGGTGGAAGAAT and the reverse primer was designed as TCATAACTTATTGTTGTACCAATACACA using N-terminal and C-terminal nucleotide sequences information of *Physaria lindheimeri* transcriptome. Since CDS sequences between *PlFAD3-1* and *PlFAD3-2* are similar, primers to clone PlFAD3-1 was designed in UTR region of *PlFAD3-1* cDNA. The forward primer for *PlFAD3-1* was GTCCCTAACTTTGAGATTAGAGATT and the reverse primer was AAGCTAAACAAAGGAAAAGGAAGA. The RT-PCR product was amplified using Ex Tag polymerase (Takara) and purified using a Purification kit (Cosmo genetech, http://www.cosmogenetech.com/). The purified PCR product was ligated into pGEMT-Easy vector (Promega, https://www.promega.com/). The universal M13 forward and reverse primers was used to confirm the sequence of *PlFAD2* and *PlFAD3-1* in Sanger DNA sequencing. Full length cDNAs sequences information were deposit to NCBI GenBank, with MW139649 for *PlFAD2*, and MW139650 for *PlFAD3-1*.
