# Supplementary material for: Transcriptome Analysis and Identification of Lipid Genes in Physaria lindheimeri, a Genetic Resource for Hydroxy Fatty Acids in Seed Oil
Source: Int J Mol Sci. 2021 Jan 6;22(2):514. doi: 10.3390/ijms22020514 (PMC7825617; doi:10.3390/ijms22020514)
Supplement: Supplementary file 1 [file ijms-22-00514-s001.zip › reiviosin ijms-1021173 Sup files_KHU and Chen/Sup file 7, Figure S7.pptx]

## Slide 1
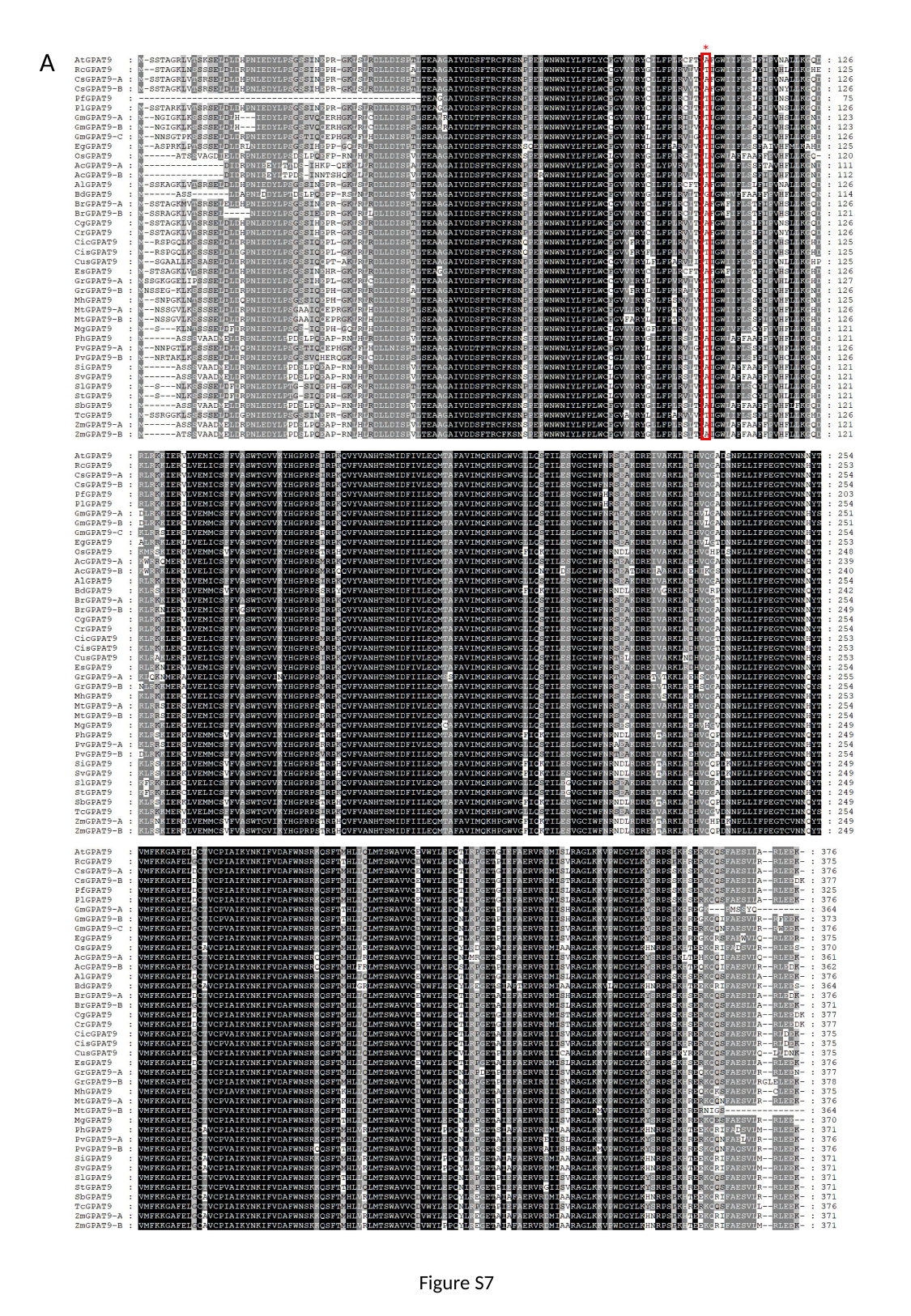

*
A
Figure S7

## Slide 2
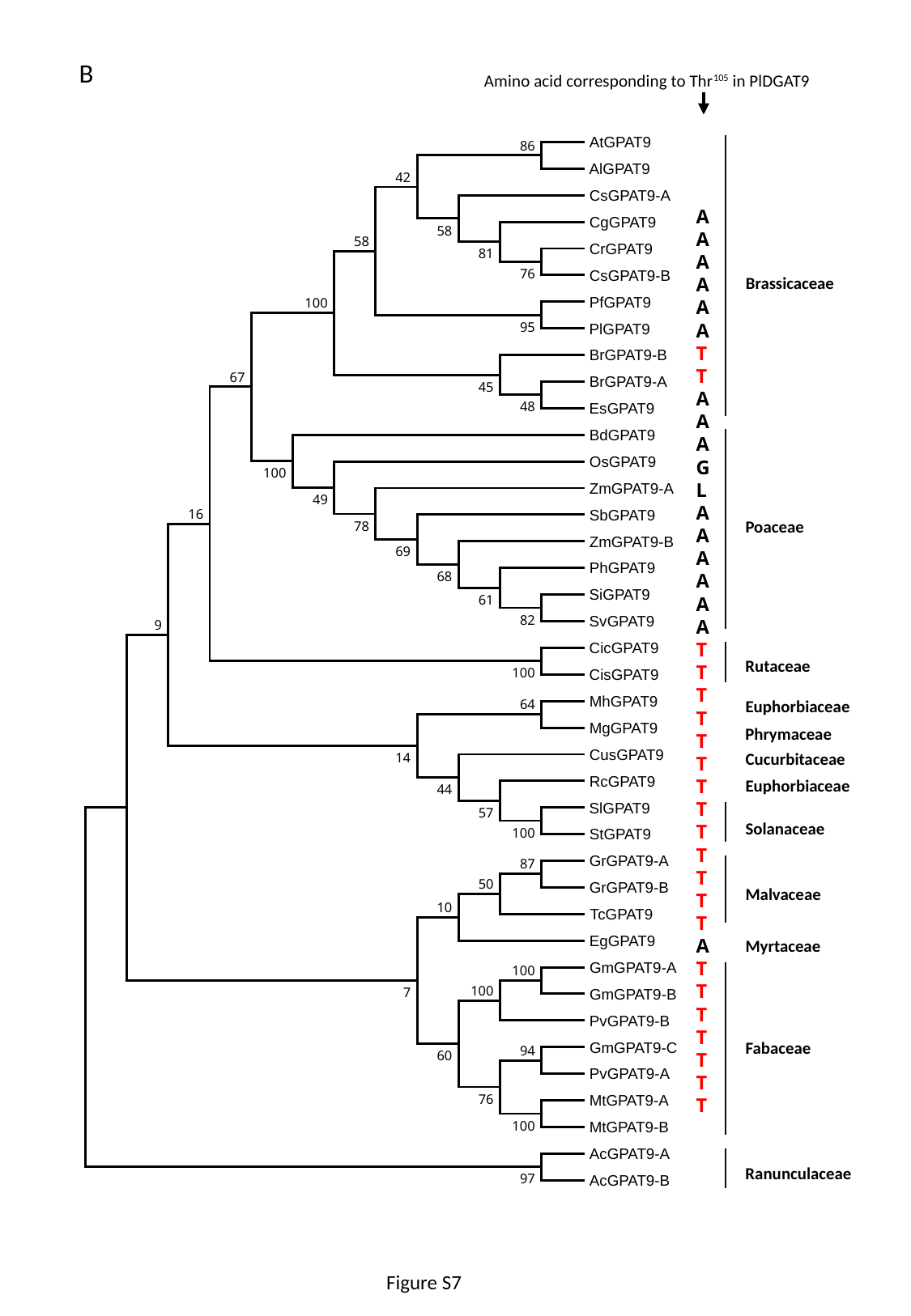

B
Amino acid corresponding to Thr105 in PlDGAT9
 AtGPAT9
86
 AlGPAT9
42
 CsGPAT9-A
 CgGPAT9
58
58
 CrGPAT9
81
76
 CsGPAT9-B
 PfGPAT9
100
95
 PlGPAT9
 BrGPAT9-B
67
 BrGPAT9-A
45
48
 EsGPAT9
 BdGPAT9
 OsGPAT9
100
 ZmGPAT9-A
49
 SbGPAT9
16
78
 ZmGPAT9-B
69
 PhGPAT9
68
 SiGPAT9
61
82
 SvGPAT9
9
 CicGPAT9
100
 CisGPAT9
 MhGPAT9
64
 MgGPAT9
 CusGPAT9
14
 RcGPAT9
44
 SlGPAT9
57
100
 StGPAT9
 GrGPAT9-A
87
50
 GrGPAT9-B
10
 TcGPAT9
 EgGPAT9
 GmGPAT9-A
100
100
7
 GmGPAT9-B
 PvGPAT9-B
 GmGPAT9-C
94
60
 PvGPAT9-A
76
 MtGPAT9-A
100
 MtGPAT9-B
 AcGPAT9-A
97
 AcGPAT9-B
AAAAAATTAAAGLAAAAAATTTTTTTTTTTTTATTTTTTT
Brassicaceae
Poaceae
Rutaceae
Euphorbiaceae
Phrymaceae
Cucurbitaceae
Euphorbiaceae
Solanaceae
Malvaceae
Myrtaceae
Fabaceae
Ranunculaceae
Figure S7

## Slide 3
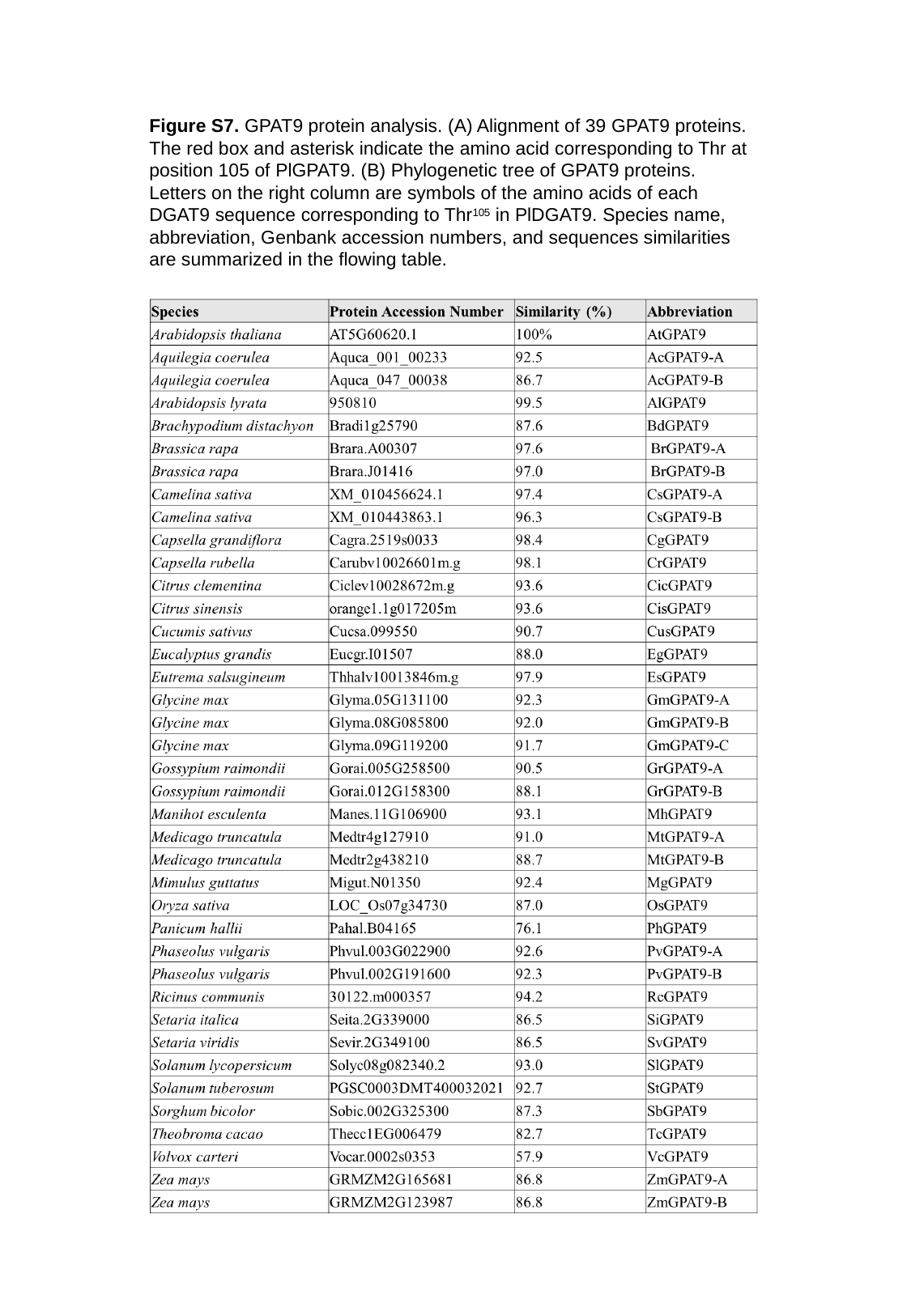

Figure S7. GPAT9 protein analysis. (A) Alignment of 39 GPAT9 proteins. The red box and asterisk indicate the amino acid corresponding to Thr at position 105 of PlGPAT9. (B) Phylogenetic tree of GPAT9 proteins. Letters on the right column are symbols of the amino acids of each DGAT9 sequence corresponding to Thr105 in PlDGAT9. Species name, abbreviation, Genbank accession numbers, and sequences similarities are summarized in the flowing table.
