# Supplementary material for: Transcriptome Analysis and Identification of Lipid Genes in Physaria lindheimeri, a Genetic Resource for Hydroxy Fatty Acids in Seed Oil
Source: Int J Mol Sci. 2021 Jan 6;22(2):514. doi: 10.3390/ijms22020514 (PMC7825617; doi:10.3390/ijms22020514)
Supplement: Supplementary file 1 [file ijms-22-00514-s001.zip › reiviosin ijms-1021173 Sup files_KHU and Chen/Sup file 8, Figure S8.pptx]

## Slide 1
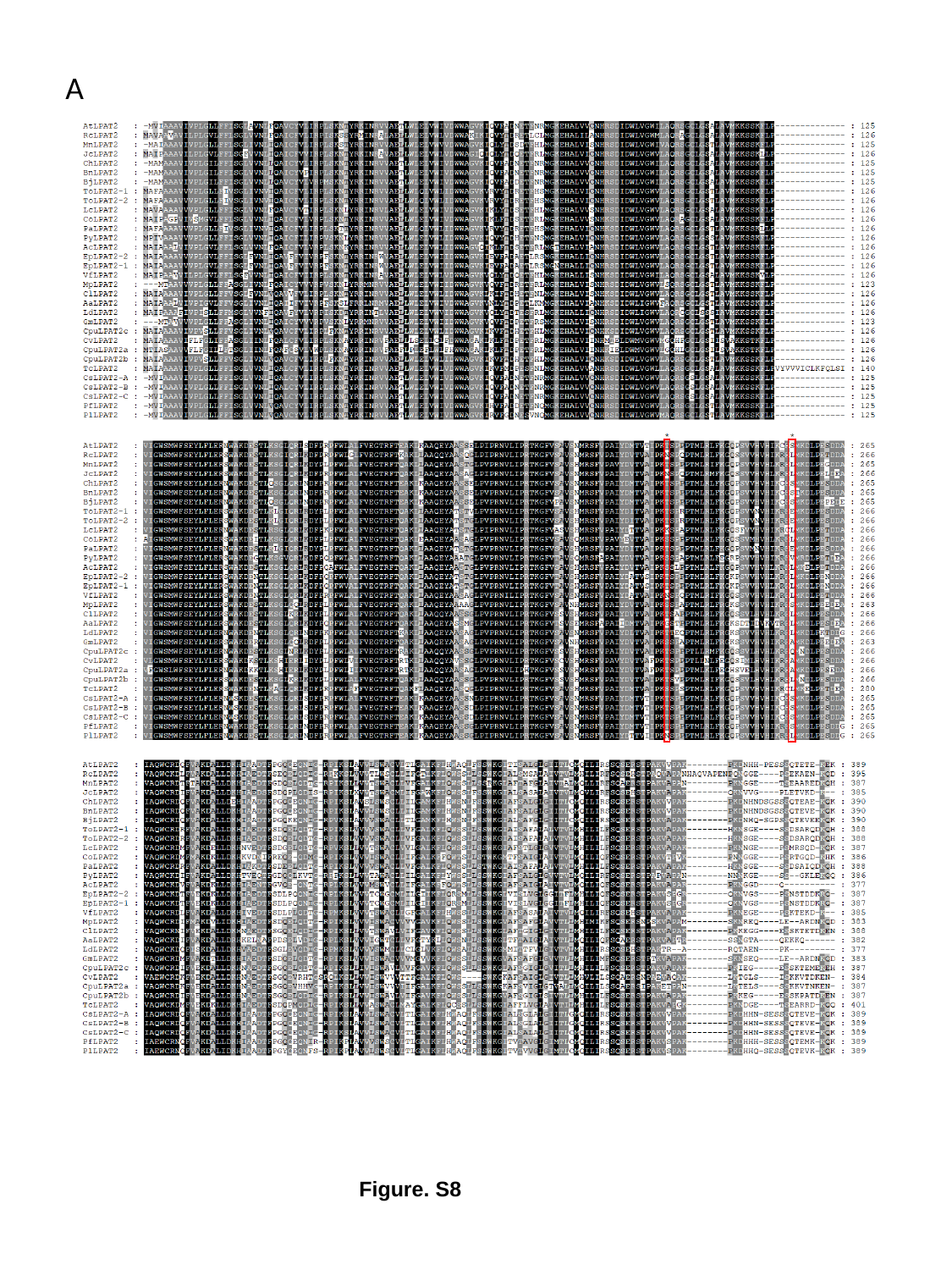

A
Figure. S8

## Slide 2
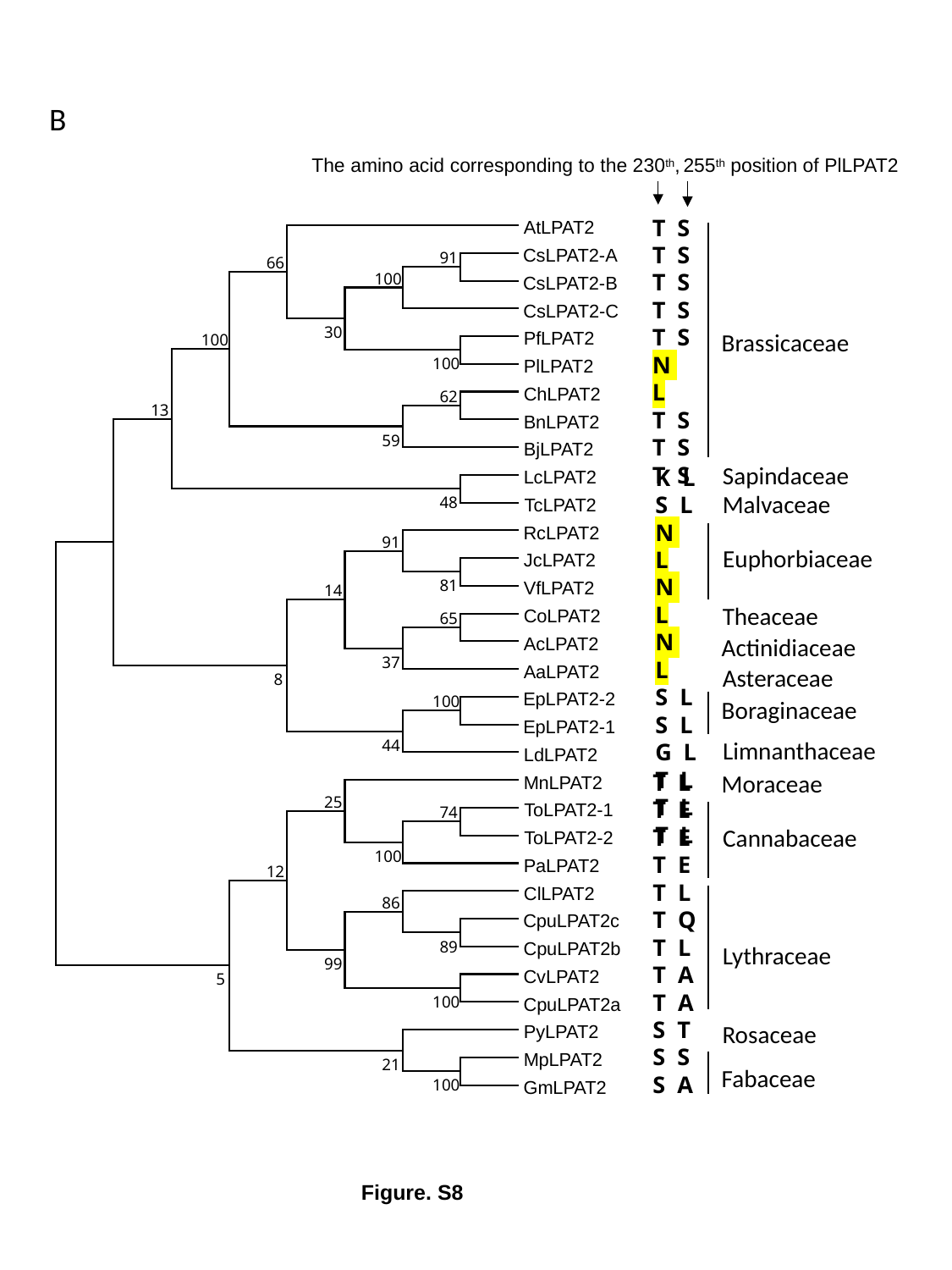

B
The amino acid corresponding to the 230th, 255th position of PlLPAT2
 AtLPAT2
 CsLPAT2-A
91
66
100
 CsLPAT2-B
 CsLPAT2-C
30
 PfLPAT2
100
100
 PlLPAT2
 ChLPAT2
62
13
 BnLPAT2
59
 BjLPAT2
 LcLPAT2
48
 TcLPAT2
 RcLPAT2
91
 JcLPAT2
81
 VfLPAT2
14
 CoLPAT2
65
 AcLPAT2
37
 AaLPAT2
8
 EpLPAT2-2
100
 EpLPAT2-1
44
 LdLPAT2
 MnLPAT2
25
 ToLPAT2-1
74
 ToLPAT2-2
100
 PaLPAT2
12
 ClLPAT2
86
 CpuLPAT2c
89
 CpuLPAT2b
99
 CvLPAT2
5
100
 CpuLPAT2a
 PyLPAT2
 MpLPAT2
21
100
 GmLPAT2
T S
T S
T S
T S
T S
N L
T S
T S
T S
Brassicaceae
Sapindaceae
K L
S L
N L
N L
N L
S L
S L
G L
T L
T L
T L
Malvaceae
Euphorbiaceae
Theaceae
Actinidiaceae
Asteraceae
Boraginaceae
Limnanthaceae
Moraceae
T L
T E
T E
T E
T L
T Q
T L
T A
T A
S T
S S
S A
Cannabaceae
Lythraceae
Rosaceae
Fabaceae
Figure. S8

## Slide 3
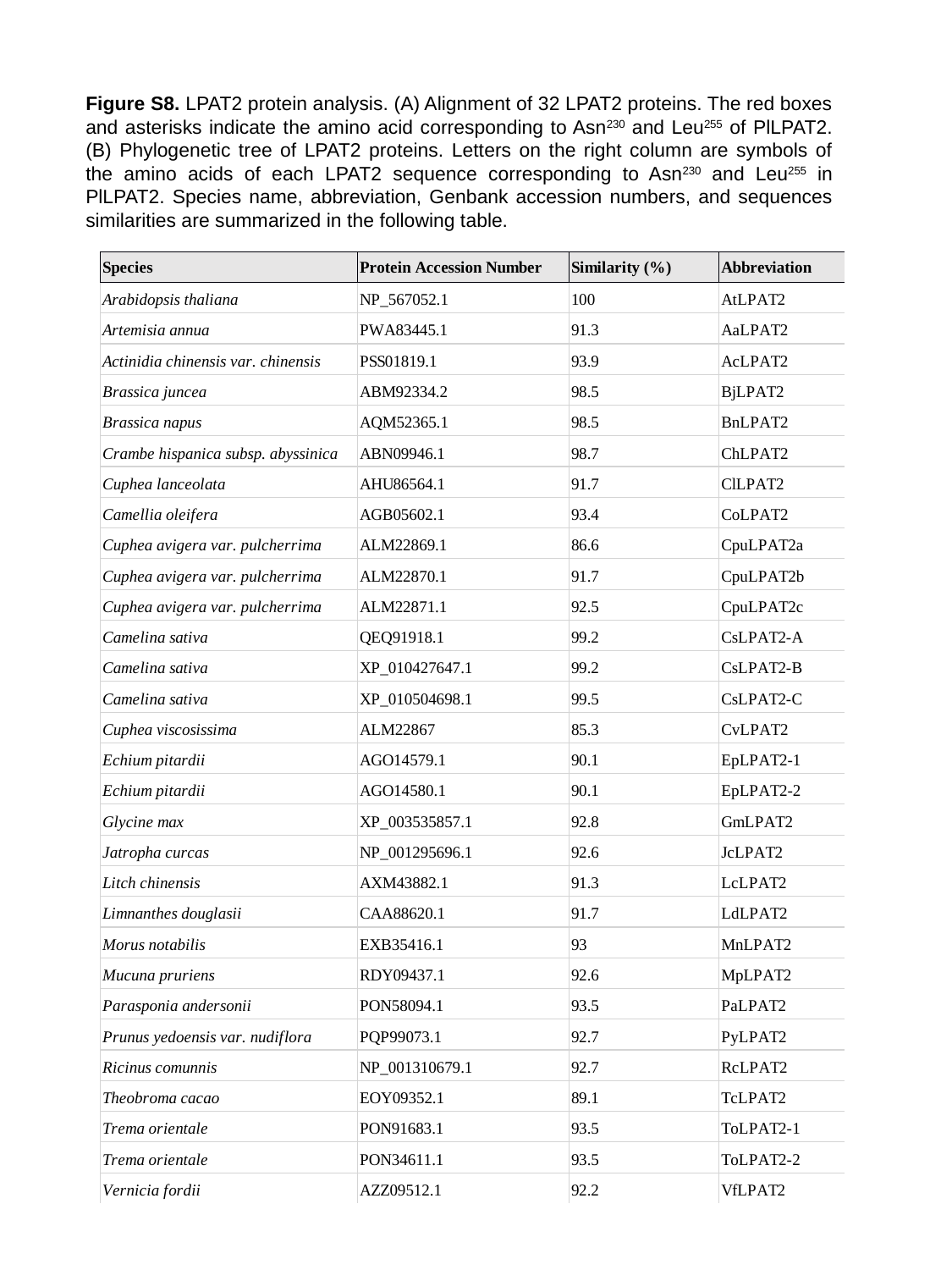

Figure S8. LPAT2 protein analysis. (A) Alignment of 32 LPAT2 proteins. The red boxes and asterisks indicate the amino acid corresponding to Asn230 and Leu255 of PlLPAT2. (B) Phylogenetic tree of LPAT2 proteins. Letters on the right column are symbols of the amino acids of each LPAT2 sequence corresponding to Asn230 and Leu255 in PlLPAT2. Species name, abbreviation, Genbank accession numbers, and sequences similarities are summarized in the following table.
