# Supplementary material for: Transcriptome Analysis and Identification of Lipid Genes in Physaria lindheimeri, a Genetic Resource for Hydroxy Fatty Acids in Seed Oil
Source: Int J Mol Sci. 2021 Jan 6;22(2):514. doi: 10.3390/ijms22020514 (PMC7825617; doi:10.3390/ijms22020514)
Supplement: Supplementary file 1 [file ijms-22-00514-s001.zip › reiviosin ijms-1021173 Sup files_KHU and Chen/Sup file 9, Figure S9.pptx]

## Slide 1
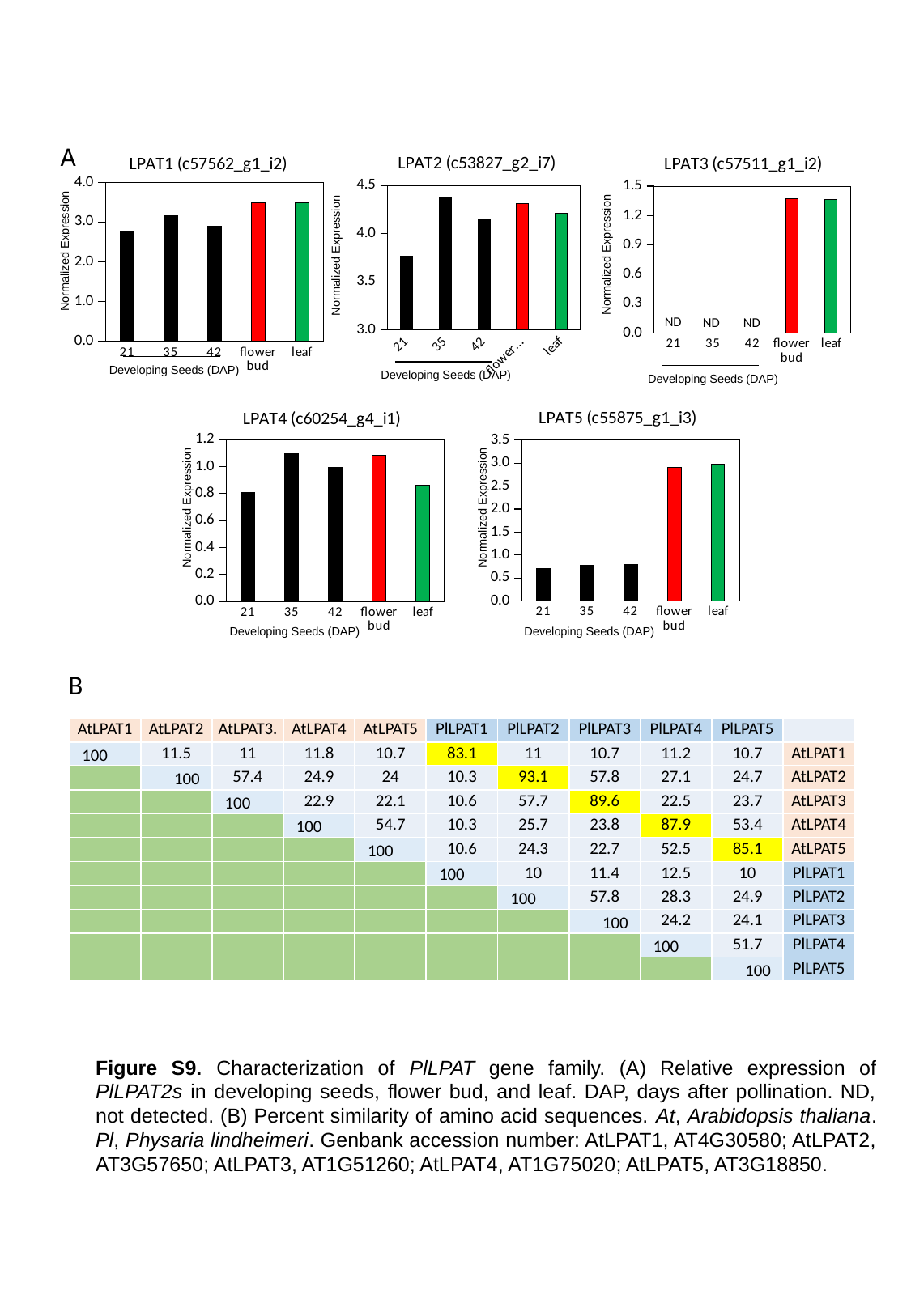

### Chart:
| Category | LPAT1 (c57562_g1_i2) |
|---|---|
| 21 | 2.75060204 |
| 35 | 3.15685998 |
| 42 | 2.89419569 |
| flower bud | 3.48246427 |
| leaf | 3.49336347 |Normalized Expression
Developing Seeds (DAP)
### Chart:
| Category | LPAT3 (c57511_g1_i2) |
|---|---|
| 21 | 0.0 |
| 35 | 0.0 |
| 42 | 0.0 |
| flower bud | 1.3739831658850965 |
| leaf | 1.3664468066500604 |Normalized Expression
Developing Seeds (DAP)
### Chart:
| Category | LPAT2 (c53827_g2_i7) |
|---|---|
| 21 | 3.76986038 |
| 35 | 4.38324263 |
| 42 | 4.14313785 |
| flower bud | 4.31452857 |
| leaf | 4.21635587 |Normalized Expression
Developing Seeds (DAP)
### Chart:
| Category | LPAT4 (c60254_g4_i1) |
|---|---|
| 21 | 0.81068845 |
| 35 | 1.09614107 |
| 42 | 0.99117822 |
| flower bud | 1.08194381 |
| leaf | 0.86399302 |Normalized Expression
Developing Seeds (DAP)
### Chart:
| Category | LPAT5 (c55875_g1_i3) |
|---|---|
| 21 | 0.71104637 |
| 35 | 0.78319338 |
| 42 | 0.79455106 |
| flower bud | 2.910406 |
| leaf | 2.97928124 |Normalized Expression
Developing Seeds (DAP)
A
ND
ND
ND
B
| AtLPAT1 | AtLPAT2 | AtLPAT3. | AtLPAT4 | AtLPAT5 | PlLPAT1 | PlLPAT2 | PlLPAT3 | PlLPAT4 | PlLPAT5 | |
| --- | --- | --- | --- | --- | --- | --- | --- | --- | --- | --- |
| 100 | 11.5 | 11 | 11.8 | 10.7 | 83.1 | 11 | 10.7 | 11.2 | 10.7 | AtLPAT1 |
| | 100 | 57.4 | 24.9 | 24 | 10.3 | 93.1 | 57.8 | 27.1 | 24.7 | AtLPAT2 |
| | | 100 | 22.9 | 22.1 | 10.6 | 57.7 | 89.6 | 22.5 | 23.7 | AtLPAT3 |
| | | | 100 | 54.7 | 10.3 | 25.7 | 23.8 | 87.9 | 53.4 | AtLPAT4 |
| | | | | 100 | 10.6 | 24.3 | 22.7 | 52.5 | 85.1 | AtLPAT5 |
| | | | | | 100 | 10 | 11.4 | 12.5 | 10 | PlLPAT1 |
| | | | | | | 100 | 57.8 | 28.3 | 24.9 | PlLPAT2 |
| | | | | | | | 100 | 24.2 | 24.1 | PlLPAT3 |
| | | | | | | | | 100 | 51.7 | PlLPAT4 |
| | | | | | | | | | 100 | PlLPAT5 |
Figure S9. Characterization of PlLPAT gene family. (A) Relative expression of PlLPAT2s in developing seeds, flower bud, and leaf. DAP, days after pollination. ND, not detected. (B) Percent similarity of amino acid sequences. At, Arabidopsis thaliana. Pl, Physaria lindheimeri. Genbank accession number: AtLPAT1, AT4G30580; AtLPAT2, AT3G57650; AtLPAT3, AT1G51260; AtLPAT4, AT1G75020; AtLPAT5, AT3G18850.
